# Comparative Genomics Reveals Sources of Genetic Variability in the Asexual Fungal Plant Pathogen *Colletotrichum lupini*

**DOI:** 10.1111/mpp.70039

**Published:** 2024-12-13

**Authors:** Joris A. Alkemade, Pierre Hohmann, Monika M. Messmer, Timothy G. Barraclough

**Affiliations:** ^1^ Department of Biology University of Oxford Oxford UK; ^2^ Calleva Research Centre for Evolution and Human Science Magdalen College Oxford UK; ^3^ Department of Crop Sciences Research Institute of Organic Agriculture (FiBL) Frick Switzerland; ^4^ Department of Biology, Healthcare and the Environment, Faculty of Pharmacy and Food Sciences Universitat de Barcelona Barcelona Spain

**Keywords:** accessory chromosomes, anthracnose, lupin, transposons

## Abstract

Fungal plant pathogens cause major crop losses worldwide, with many featuring compartmentalised genomes that include both core and accessory regions, which are believed to drive adaptation. The highly host‐specific fungus *Colletotrichum lupini* greatly impacts lupin (*Lupinus* spp.) cultivation. This pathogen is part of clade 1 of the *C. acutatum* species complex and comprises four genetically uniform, presumably clonal, lineages (I–IV). Despite this, variation in virulence and morphology has been observed within these lineages. To investigate the potential sources of genetic variability in this asexual fungus, we compared the genomes of 16 *C. lupini* strains and 17 related *Colletotrichum* species. Phylogenomics confirmed the presence of four distinct lineages, but further examination based on genome size, gene content, transposable elements (TEs), and deletions revealed that lineage II could be split into two groups, II‐A and II‐B. TE content varied between lineages and correlated strongly with genome size variation, supporting a role for TEs in genome expansion in this species. Pangenome analysis revealed a highly variable accessory genome, including a minichromosome present in lineages II, III, and IV, but absent in lineage I. Accessory genes and effectors appeared to cluster in proximity to TEs. Presence/absence variation of putative effectors was lineage‐specific, suggesting that these genes play a crucial role in determining host range. Notably, no effectors were found on the TE‐rich minichromosome. Our findings shed light on the potential mechanisms generating genetic diversity in this asexual fungal pathogen that could aid future disease management.

## Graphical abstract

Comparative genomics of the lupin pathogen *Colletotrichum lupini* reveals low SNP diversity but highlights a dynamic accessory genome, including a variable accessory chromosome and diverse transposable element landscape.

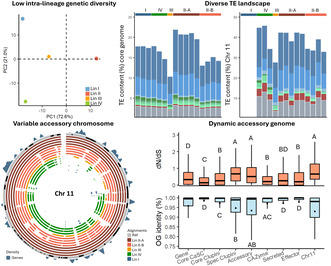

## 1 Introduction

The interaction between fungi and plants is ancient and spans a continuum from mutualistic to parasitic and from epiphytic to endophytic (Naranjo‐Ortiz and Gabaldón 2020). To facilitate successful colonisation of a host, fungi secrete effector molecules to manipulate the plant immune system or ward off other microbes (Snelders, Rovenich, and Thomma 2022; Cook, Mesarich, and Thomma 2015; Plett and Martin 2015; Redkar et al. 2022). For pathogenic fungi, there is strong evolutionary pressure to prevent host recognition (Möller and Stukenbrock 2017). Across eukaryotes, rapid evolution is generally enabled by sexual reproduction, which generates new gene combinations in a population (Tellier, Moreno‐Gámez, and Stephan 2014). Yet, sexual reproduction in fungi is often rare, with one fifth of described species being presumed predominantly asexual (Naranjo‐Ortiz and Gabaldón 2020; Taylor, Jacobson, and Fisher 1999), including important plant pathogens such as *Fusarium oxysporum* (McTaggart et al. 2021; Ordonez et al. 2015), *Magnaporthe oryzae* (Latorre et al. 2023) and *Verticillium dahliae* (de Jonge et al. 2013). Understanding how they generate the variability required to keep pace with coevolving host immune defences is important for improving disease management.

Many filamentous plant pathogens have segmented genomes, often referred to as the two‐speed genome (Dong, Raffaele, and Kamoun 2015), with effector genes mostly localised within highly variable repeat‐rich regions, enabling fast diversification (Torres, Thomma, and Seidl 2021; van Westerhoven et al. 2024; Sánchez‐Vallet et al. 2018). Pangenome analyses have shown that effector gene repertoires greatly fluctuate between closely related lineages and are often crucial for determining host range (Badet et al. 2020; van Dam et al. 2016; Le Naour‐Vernet et al. 2023). Alongside diverse effector repertoires, many fungal genomes exhibit structural variation within species, such as the occurrence of large duplication and deletion events and varying numbers of accessory chromosomes (ACs; Todd, Forche, and Selmecki 2017; Möller and Stukenbrock 2017), highlighting the diversification potential of (asexual) plant‐pathogenic fungi.

*Colletotrichum* species are notorious plant pathogens, causing devasting pre‐ and postharvest disease in numerous crops (Cannon et al. 2012). *Colletotrichum* is traditionally recognised as asexual (Cannon et al. 2012), but sexual reproduction has been observed in a few rare cases (Damm et al. 2012; Wilson et al. 2021; Rogério et al. 2022). Despite the rarity of sexual recombination, *Colletotrichum* species are highly diverse in both lifestyle and host range (Talhinhas and Baroncelli 2021). The mechanisms behind the emergence of this diversity are not yet fully understood (da Silva et al. 2020) but could include the mechanisms outlined above. For example, the evolution of ACs seems to play a significant role in host adaptation by serving as vehicles for horizontal gene transfer (HGT) to acquire novel pathogenicity genes (Wang et al. 2023). In *C. lentis* and *C. graminicola* , ACs were shown to be crucial to achieve full virulence in their respective hosts (Bhadauria et al. 2019; Ma et al. 2023). Transposable elements (TEs) were shown to generate genome variability and diversity in *C. higginasum* (Tsushima et al. 2019), a pathogen of several brassica species. Furthermore, TE activity in various *Colletotrichum* species influenced the gain and loss of gene families, which contributed to the adaptation of different lifestyles (Gan et al. 2016), such as host jumps from monocot to eudicot plants (Baroncelli et al. 2024).

Lupin anthracnose, caused by *C. lupini* , is hampering sustainable cultivation of the high‐quality protein crops of white ( *Lupinus albus* ), blue ( *L. angustifolius* ), yellow ( *L. luteus* ), and Andean lupin ( *L. mutabilis* ). These crops have high potential to provide a locally produced alternative to soybean in temperate regions. *C. lupini* belongs to clade 1 of the *C. acutatum* species complex (CaSC), which harbours many devastating plant pathogens (Damm et al. 2012). In previous work, 67 *C. lupini* isolates, collected from lupin production areas across the world, were characterised through 3D‐RAD sequencing (Alkemade et al. 2023). Population genetics showed that, like many other CaSC species (Damm et al. 2012), *C. lupini* seems to be asexual, with four (I–IV) distinct and genetically uniform lineages displaying low admixture and high divergence (Alkemade et al. 2023). Despite the high genetic uniformity, differences in virulence and morphology were observed amongst and within those four lineages (Alkemade et al. 2021). All four lineages were present in the Andes region of South America, which is the presumed centre of origin of *C. lupini* (Riegel et al. 2010) and other CaSC clade 1 species (Bragança et al. 2016). Isolates found outside South America after 1990 all belong to lineage II, which contains strains that are highly aggressive on white and Andean lupin (Alkemade et al. 2021). The escape and global spread of a genetically uniform lineage, as seen for *C. lupini* , is typical for invasive fungi (Gladieux et al. 2016) and has been observed for numerous plant pathogens such as *Fusarium odoratissimum* on banana (van Westerhoven et al. 2022) and *M. oryzae* on wheat (Latorre et al. 2023). Yet, the potential genetic basis for differences within and between lineages in *C. lupini* remains unexplored.

In this study, we investigate inter‐ and intralineage genetic variability within *C. lupini* by sequencing the genomes of 16 strains, collected from five continents and 10 countries, representing all four lineages, and comparing them to each other and to other CaSC species. We quantify the extent to which genome structure, TE landscape, the gain or loss of effectors, and selection on different gene categories, in turn, contribute towards genetic diversity within and between *C. lupini* lineages. The findings should provide insights on the evolution and genetic variability of a putatively asexual fungal plant pathogen.

## 2 Results

### 2.1 Genomic Diversity Within the *C. acutatum* Species Complex

**FIGURE 1:**
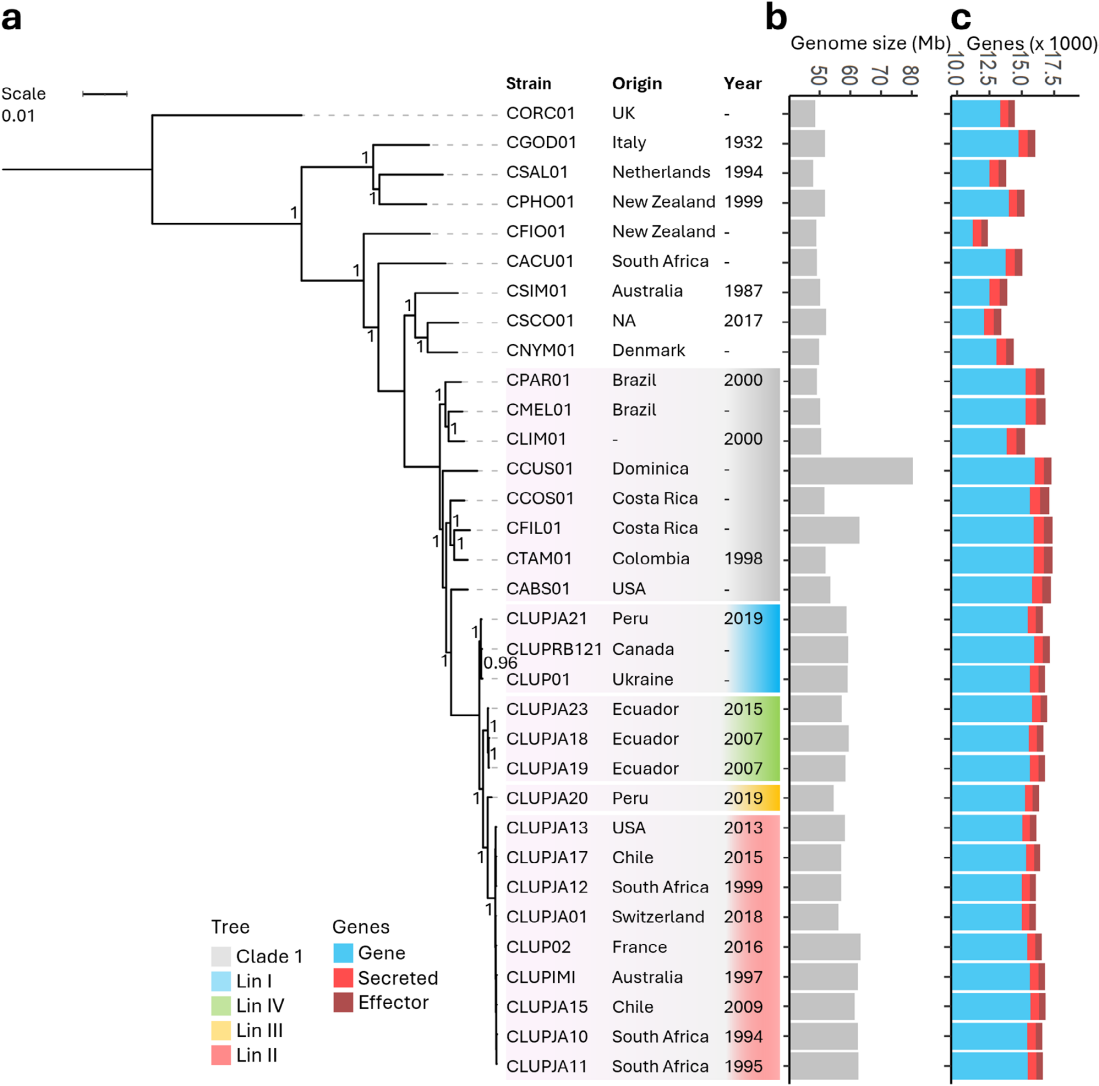
Phylogenomic tree, genome size and gene content of the *Colletotrichum acutatum* species complex (CaSC). (a) Tree constructed based on 6350 single‐copy orthologue genes (3,520,830 amino acids) using FastTree. Colours in the tree indicate *C. lupini* lineage or CaSC clade 1. Bootstrap support values are given at each node. Columns indicate strain name, origin, and year of collection. (b) Genome size (Mb) of CaSC species. (c) Predicted (secreted) gene and effector count of CaSC species. Colours indicate gene (blue), secreted (red), or effector (dark red).

**FIGURE 2:**
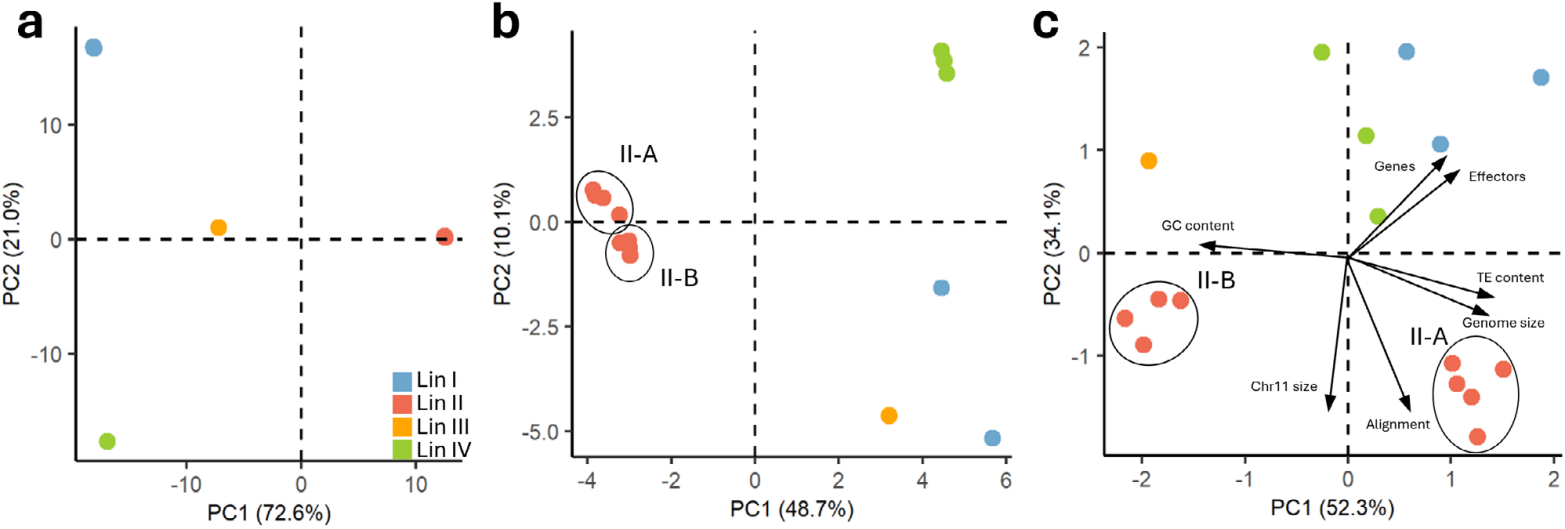
Global genetic structure of 16 *Colletotrichum lupini* strains based on (a) 310,498 single‐nucleotide polymorphisms (SNPs). (b) Presence/absence matrix of transposable element (TE) insertions. (c) Overall genome statistics: genome size, GC content, gene content, effector content, chromosome 11 size, TE content, and proportion of alignment to CLUP02. Principal components 1 and 2 are plotted. Blue indicates lineage I, red: lineage II, orange: lineage III and green: lineage IV. Lineage II‐A and B are indicated with a circle.

To study *C. lupini* evolution, 14 strains that were collected from across the globe and represented all four lineages (Figure S1) were sequenced and assembled using short‐read data at an average of 141× coverage (Table S1). Two publicly available *C. lupini* long‐read assemblies (CBS 109225 and IMI 504893, referred to as CLUP01 and CLUP02, respectively) were included as well (Baroncelli et al. 2021; Baroncelli et al. 2024). Seventeen publicly available genomes of species representing the five different clades of the CaSC were included to assess the genetic diversity of *C. lupini* in relation to the CaSC (Baroncelli et al. 2024). Although short‐read assemblies were more fragmented (484–2499 contigs) than long‐read assemblies (11–116 contigs), all *C. lupini* assemblies contained at least 96% of the single‐copy BUSCO genes (Table S1), indicating nearly complete coverage of the conserved genes. *C. lupini* genome sizes ranged between 54.5 and 63.4 Mb (Figure 1b), corresponding to total K‐mer counts (Table S1). The average *C. lupini* genome size of 59.2 Mb was larger than other CaSC species, except for *C. filicis* (62.96 Mb) and the very large *C. cuscutae* (80.45 Mb). De novo gene annotation on all *C. lupini* genomes resulted in a predicted gene count ranging between 16,077 and 17,181 (Figure 1c). The number of predicted secreted proteins within *C. lupini* ranged from 998 to 1107 and predicted effectors ranged from 418 to 480. Average predicted gene content for *C. lupini* (16,600) was similar to clade 1 (16,912) but higher than other CaSC species (14,294), whereas predicted effectors were lowest for *C. lupini* (497), followed by CaSC (562) and other clade 1 species (669; Figure S2). To assess genetic diversity, a phylogenomic tree was constructed from 6350 single‐copy orthologue genes (3,520,830 amino acids [AA], PID = 94.9%). As expected, *C. lupini* was placed in clade 1 and consisted of four distinct but highly uniform lineages (Figure 1a). These four highly uniform lineages were also observed with a principal component analysis (PCA) based on 310,498 single‐nucleotide polymorphisms (SNPs; Figure 2a). However, within lineage (Lin) II clear differences in genome sizes were observed (Figure 1b).

### 2.2 Substantial Genome Variation Within *C. lupini*

**FIGURE 3:**
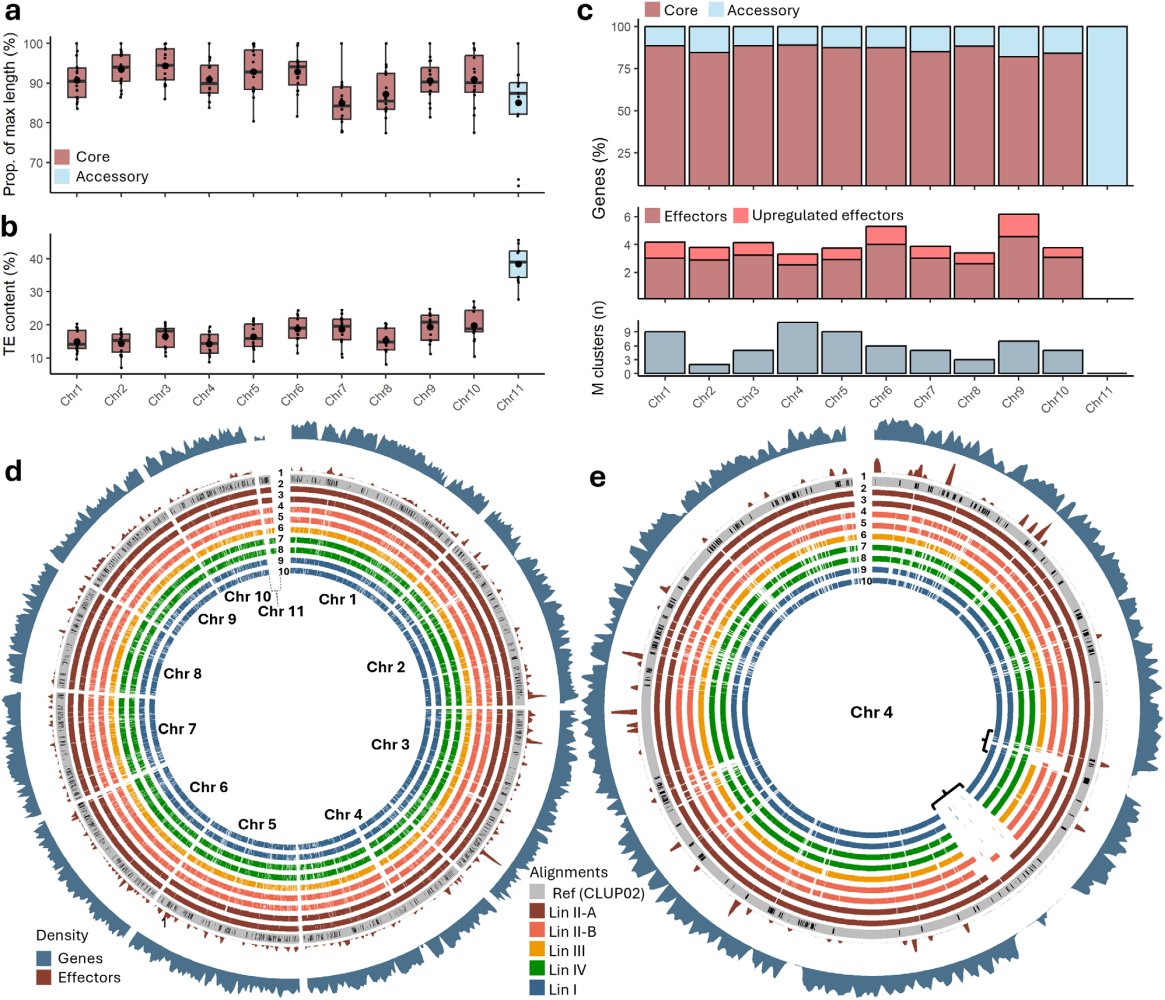
Genome variability of *Colletotrichum lupini*. (a) Chromosome length variation expressed as the percentage of the maximum observed length. Lineage I strains are excluded for chromosome 11. (b) Transposable element (TE) content (%) for each chromosome. (c) Top, percentage of core (red), and accessory (blue) genes per chromosome. Middle, percentage of upregulated (red) and stably expressed (dark red) effectors per chromosome. Bottom, number of metabolic gene clusters per chromosome. (d) Whole‐genome alignments with gene (blue) and effector (red) abundance (min = 0.2, max = 3.5/kb). (e) Chromosome 4 alignment with gene and effector abundance (min = 0, max = 2.5/kb), gaps of 350 and 100 kb are indicated. Within alignment plots: 1: reference genome CLUP02 with annotated transposons, 2: CLUPJA10, 3: CLUPJA15, 4: CLUPJA01, 5: CLUPJA17, 6: CLUPJA20, 7: CLUPJA18, 8: CLUPJA23, 9: CLUP01, and 10: CLUPJA21.

To identify core (present in all strains) and accessory (present in some strains) chromosomes, Ragtag assemblies based on reference CLUP02 were used. Ten core and one accessory chromosome (AC), absent in Lin I, were identified in *C. lupini* . Core chromosomes showed an average size variation of 9.08%, with the highest variation of 22.54% observed for chromosome 8 (Figure 3a). AC 11 size ranged from 0.34 to 0.52 Mb, indicating an average variation of 14.89%, with the highest deviation compared to CLUP02 of 35.87% seen in CLUPJA19. Comparing genome sizes within *C. lupini* showed a variation of 5.58% within Lin II, compared to a variation of 0.5% for Lin I and 1.85% for Lin IV (Figure 1, Figure S3). Similarly, there was greater GC content (Lin II = 2.96%, Lin I = 0.24%, and Lin IV = 0.90%), gene content (Lin II = 2.25%, Lin I = 1.83%, and Lin IV = 0.92%), and effector content variation (Lin II = 4.75%, Lin I = 2.02%, and Lin IV = 0.73%) in Lin II compared to the other lineages. When plotting these variables, together with AC 11 size, TE content, and proportion of alignment to CLUP02 in a PCA, two distinct Lin II groups were identified (Figure 2c). These groups will be referred to as Lin II‐A, including CLUP02, ‐IMI, ‐JA10, ‐JA11, and ‐JA15, and Lin II‐B, including CLUPJA01, ‐JA12, ‐JA13, and ‐JA17. The total size of unmapped contigs ranged between 22.6 and 1165 kb and had a GC content ranging between 23.1% and 54.7%, with an average of 30.8%. The average size of unmapped contigs for Lin II‐A was 67 kb, 447 kb for II‐B, 400 kb for I, 607 kb for III, and 719 kb for IV (Figure S3).

**FIGURE 4:**
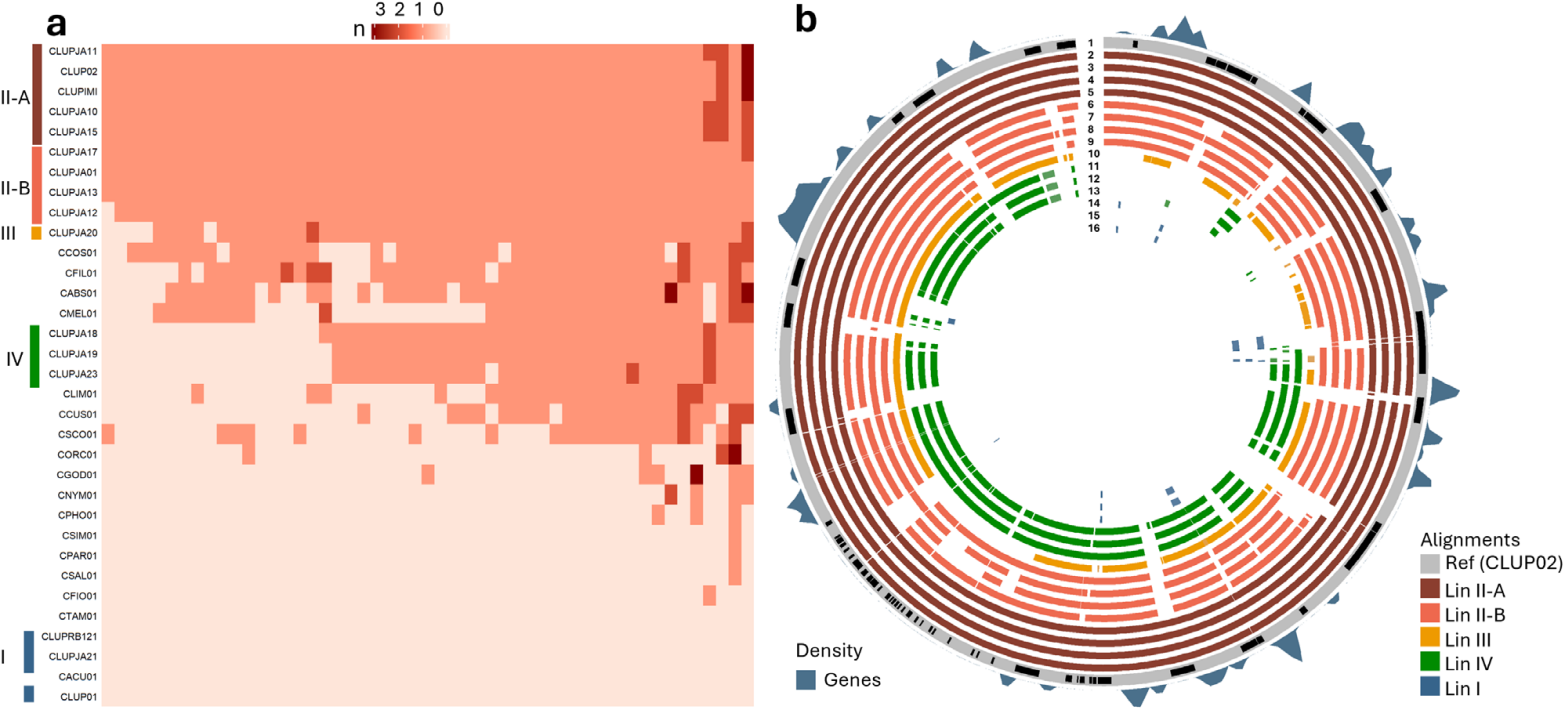
Overview of accessory chromosome 11: (a) orthogroups (columns) shared amongst *Colletotrichum acutatum* species complex (CaSC) species (rows). (b) Whole‐genome alignment ( > 80%) with gene (blue) abundance (min = 0, max = 0.3/kb). From outer ring to inner: 1: reference CLUP02 with annotated transposons shown in black, 2: CLUPIMI, 3: CLUPJA10, 4: CLUPJA11, 5: CLUPJA15, 6: CLUPJA01, 7: CLUPJA12, 8: CLUPJA13, 9: CLUPJA17, 10: CLUPJA20, 11: CLUPJA18, 12: CLUPJA19, 13: CLUPJA23, 14: CLUP01, 15: CLUPJA21, and 16: CLUPRB121. Total length of outer ring is 0.52 Mb.

Deletions were identified through whole‐genome alignments ( > 80% identity) against the reference genome CLUP02 (Figure 3d). Two large deletions, ranging between 350 and 100 kb, were found in gene‐poor regions of chromosome 4 (Figure 3e), and another 200 kb deletion was observed in a gene‐poor region of chromosome 2 (Figure 3d). AC 11 showed the most variability, being completely absent in Lin I strains, with Lin IV strains only aligning to 70.5% and Lin III strain CLUPJA20 aligning to 82.2% of the reference (Figure 4b). Substantial diversity was noted within Lin II, where group A aligned to 90% and group B aligned to 86.5% of AC 11 of CLUP02. The observed deletions matched strain relatedness, with Lin I, III, and IV showing an average alignment of 97.7%. Within Lin II, group A aligned to 99.83% of the reference genome, while group B aligned to 98.91%. Synteny analysis between long‐read assemblies CLUP01 (Lin I) and CLUP02 (Lin II) revealed high synteny apart from two major rearrangements between chromosomes 5 and 7 (621 kb) and chromosomes 2 and 6 (209 kb) in CLUP02 and CLUP01, respectively (Figure S4).

### 2.3 Distinct Lineage‐Specific TE Landscapes

**FIGURE 5:**
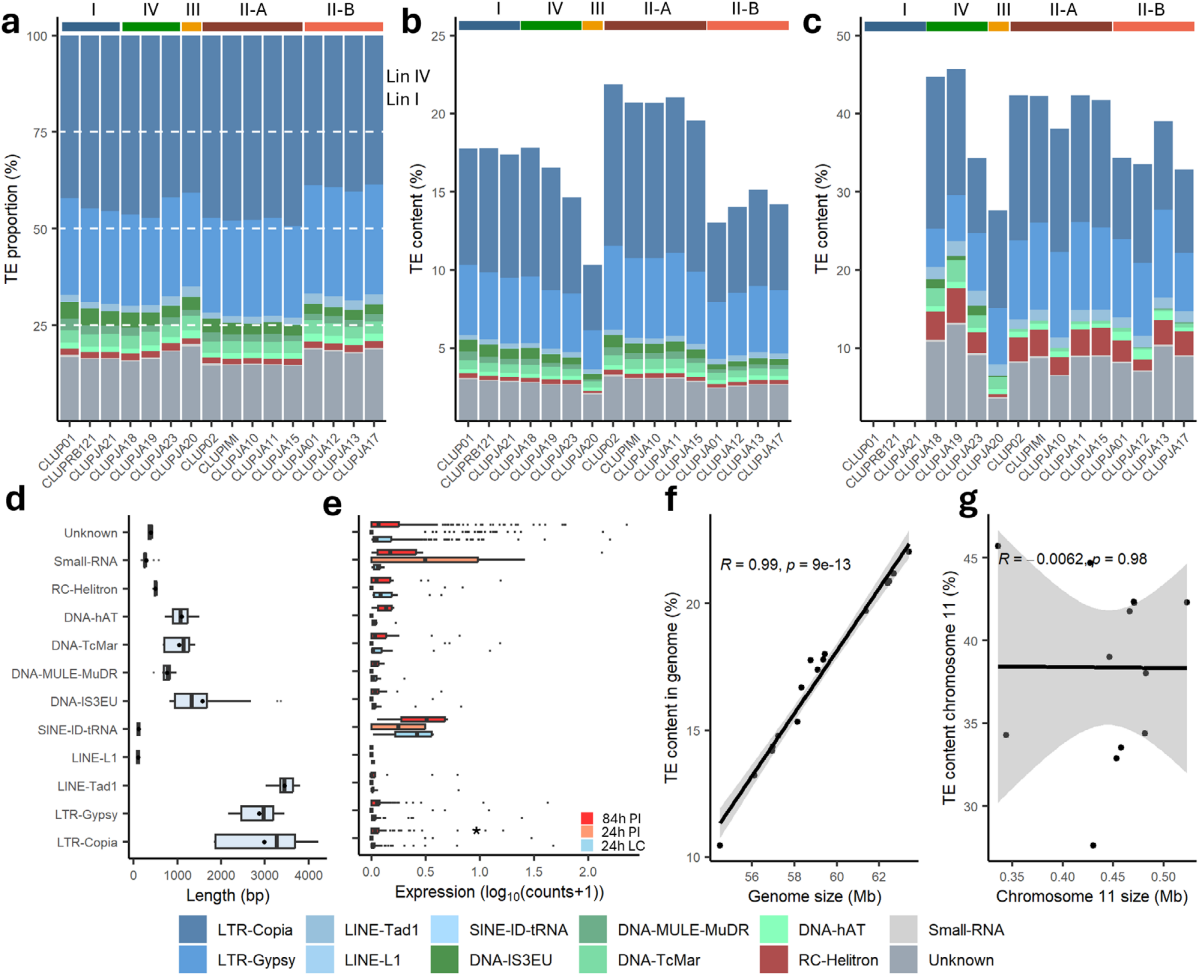
Transposable element (TE) landscape of *Colletotrichum lupini*. (a) Relative frequency of TE superfamilies across all genomes with 100% referring to the total TE content of the respective genome. (b) Contribution of TE superfamilies to core genome size. (c) Contribution of TE superfamilies to accessory chromosome 11 size. (d) TE superfamily length in bp, black dots indicate means. (e) TE de‐repression shown as expression at 24 h in liquid culture (LC), 24 and 84 h after white lupin inoculation (PI), asterisk (*) indicates significant difference between 24 h in LC and infection treatment (Kruskal–Wallis, *p* < 0.05). (f) Correlation (Pearson) of TE content (%) to genome size. (g) Correlation of TE content (%) to chromosome 11 size. Bottom: transposon superfamily legend.

TEs were predicted based on a consensus repeat library of known *Colletotrichum* repeats (Dallery et al. 2017), de novo predicted repeats from CLUP02 and CLUP01, and a combined DFAM and Repbase library. Total TE content ranged from 9.79% (CLUPJA20) to 20.59% (CLUP02, Figure 5b). The highest TE content was observed in AC 11 and ranged from 26.26% (CLUPJA20) to 42.30% (CLUPJA19; Figures 3b and 5c). High variability in TE content was observed within Lin II, with an average TE content of 19.68% for II‐A and 13.38% for II‐B (Figure S3). Total TE content (%) was strongly correlated with genome size (*r* = 0.99, *p* = 5e−13; Figure 5f) and core chromosomes (*r* = 0.94 to *r* = 0.7; Figure S5), whereas AC 11 TE content did not correlate with its size (*r* = −0.024, *p* = 0.94; Figure 5g). The long terminal repeat (LTR)‐retrotransposons Copia and Gypsy were most common, representing on average 44.98% and 25.48% of the total TE content, respectively (Figure 5a). Unknown repeats were the third most common, representing 16.57% of the total TE content, and the most abundant DNA transposon, IS3EU, represented 3.15%. LTR‐Copia (*r* = 0.98, *p* = 2e−12) and unknown repeat content (*r* = 0.97, *p* = 2.7e−10) were highly correlated with total genome size, whereas LTR‐Gypsy content was not (*r* = 0.43, *p* = 0.1; Figure S5), indicating that LTR‐Copia has the highest impact on observed genome size variations. The long interspersed nuclear elements (LINE)‐Tad1 transposons were on average the largest (3495 bp), followed by the LTR retrotransposons Copia (2945 bp) and Gypsy (2831 bp; Figure 5d). On AC 11, DNA transposon IS3EU was only present in Lin III and IV, and rolling circle (RC) helitrons were almost absent in CLUPJA20 (Lin III) representing only 0.22% compared to an average of 1.65%. To further analyse TE diversity within *C. lupini* , all genomes were screened for TE insertions, which were classified as present (1) or absent (0) in the reference genome. TE insertions resembled a normal distribution, with most insertions being present in half of all the strains. Only 5% of the insertions was present in only one of the isolates (Figure S6). A PCA was created based on the presence/absence matrix of TE insertions, showing the four expected lineages but with a higher interlineage variability than was shown with SNPs (Figure 2b).

### 2.4 Pangenome Analysis Reveals Dynamic Accessory Genome

Despite previous observations of limited within‐lineage genetic variation from SNP data (Alkemade et al. 2023), variation in accessory genes/chromosomes can generate genetic variation within a species. To analyse gene‐content diversity, all 265,271 predicted protein‐coding genes across the 16 *C. lupini* strains were grouped into 17,535 orthologous groups (OGs) based on protein homology. The pangenome included 14,107 (80.45%) core groups, with 13,004 (74.2%) consisting of single‐copy orthologous genes (Figure S7). Only one core group had genes with a copy number above 10, and only three had more than 5 (Figure S8). The remaining 3426 (19.54%) were considered accessory, shared among some but not all strains. Only two groups, containing six genes in total, were found in a single genome (Table S2).

When compared with the other CaSC genomes, 6350 single‐copy orthologous gene groups were shared across all species, representing core CaSC genes, while 1340 OGs were unique to *C. lupini* . Amongst the 593 OGs containing 7951 genes identified as effectors, 358 (60.37%) were part of the core genome, and the rest were accessory (Figure S7). A total of 11,087 carbohydrate‐active enzymes (CAZymes) were identified, which clustered into 707 OGs. Of these, 4598 (41%) were secreted, and a total of 586 CAZyme orthogroups (82.9%) were conserved among all *C. lupini* genomes (Figure S7). Most of the CAZymes could be grouped in five different families, with the biggest family being glycoside hydrolases (GH), representing 52.5% and approximately 363 genes per genome (Figure S9). Core gene copy number variation showed that South American strains clustered together, whereas accessory, CAZyme, and effector OGs conformed to previously described lineages (Figure S8).

**FIGURE 6:**
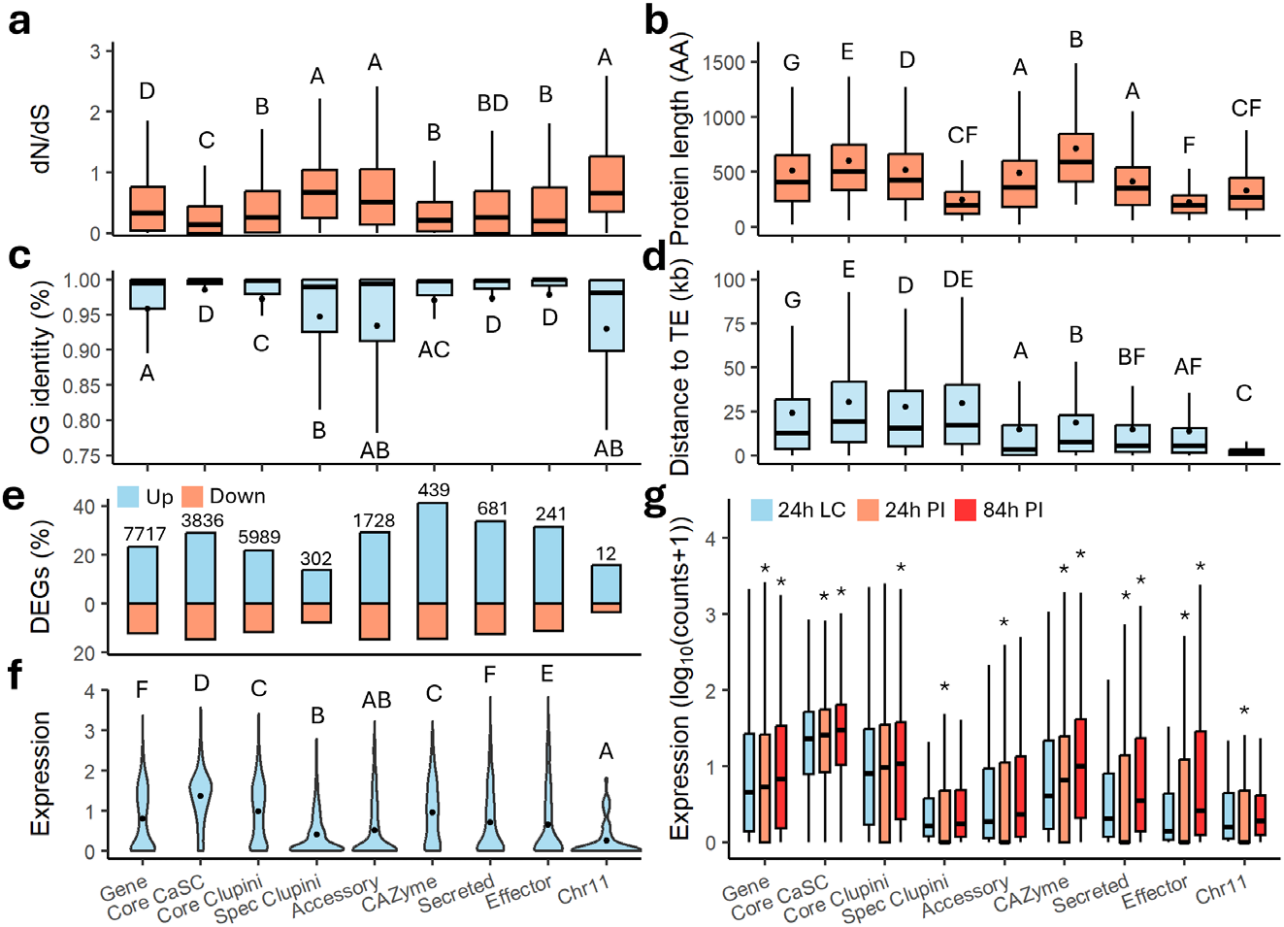
Evolutionary dynamics of different gene categories. (a) Sequence conservation estimated by the number of nonsynonymous and synonymous substitutions (dN/dS) values. (b) Percent identity given by the multiple protein sequence alignments for each orthogroup. (c) Protein length in amino acids (AA). (d) Distance (kb) to closest transposable element (TE), mapped on reference genome CLUP02. (e) Differentially expressed genes (DEGs), numbers indicate total amount of DEGs. (f) Overall expression of CLUP02 genes per category. (g) Expression of CLUP02 genes at 24 h in liquid culture (LC), 24 and 84 h after inoculation (PI), asterisks (*) indicate significant difference between 24 h in LC and infection treatment (Kruskal–Wallis, *p* < 0.05). Uppercase letter within plots indicate significant differences between gene categories (Dunns's test, *p* < 0.05). Big black dots in (b, c, d and f) indicate means.

Higher protein conservation, expressed as OG identity, was observed for effectors (97.8%), CAZymes (97.0%), and secreted genes (97.3%) compared to *C. lupini* core (96.9%) and randomly selected genes (95.8%; Figure 6c). Lowest conservation was observed for *C. lupini* ‐specific (94.7%), accessory (93.4%), and AC 11 (93.0%) genes. Additionally, *C. lupini* ‐specific (dN/dS = 0.67 [median], 250 AA), AC 11 (dN/dS = 0.66, 332 AA), and accessory (dN/dS = 0.51, 489 AA) genes appear to evolve under relaxed selective pressure and are shorter compared to core CaSC (dN/dS = 0.14, 601 AA), core *C. lupini* (dN/dS = 0.26, 521 AA), and randomly selected genes (dN/dS = 0.32, 513 AA; Figure 6a,b).

When plotted on the reference genome, effectors were not dispersed equally across the genome but were segmented in effector‐rich but gene‐poor regions (Figure 3d,e), showing a distribution coefficient of variation (CV) of 1.25 compared to a CV of 0.29 for all genes (Figure S10). Despite the high protein conservation observed in effectors, CAZymes, and secreted genes, they tend to cluster near transposable elements (TEs). Their average distances to the nearest TE and the proportions of genes within 10 kb of a TE are 14 kb (65%), 19 kb (57%), and 15 kb (64%), respectively (Figure 6c, Figure S11). This is closer than core CaSC genes (30 kb, 32%) and randomly selected genes (24 kb, 45%) but similar to accessory genes (15 kb, 67%). Genes on AC 11 are much closer, with an average distance of 2 kb and all genes within 10 kb of a TE. Whole‐genome selective sweep analysis across the 16 *C. lupini* isolates indicated 128 regions that have been subjected to positive selection (composite‐likelihood ratio [CLR] > 10; Figure S12). These regions, with the majority (95%) overlapping with TEs, contained 2.81% of accessory, 2.56% of effector, and only 1.86% of CaSC core genes. Across all lineages no selective sweep signatures were found on AC 11. Within Lin I, II, and IV, no selective sweeps were found at all, suggesting the absence of intralineage recombination. These results indicate that regions containing effector, accessory or *C. lupini* ‐specific genes tend to serve as hotspots for diversification.

Expression per gene category was based on data of CLUP02 growing for 24 h in liquid culture and 24 and 84 h after infection of white lupin ( *L. albus* ; Dubrulle et al. 2020). Analysis of differentially expressed genes (DEGs) showed that CAZyme (40.22%), secreted (33.72), effector (31.42%), and accessory (29.3%) genes had highest fractions of upregulated genes, in contrast to *C. lupini* core (21.8%), AC 11 (15.79%), and *C. lupini‐*specific (13.76%) genes (Figure 6e). Average expression (log_10_[normalised counts +1]) was highest for CaSC core genes (1.33), followed by *C. lupini* core genes (0.93; Figure 6f). Expression was lowest for accessory (0.47), *C. lupini* ‐specific (0.37), and AC 11 genes (0.23), and decreased in the early infection stage (Figure 6g). CAZymes showed an overall expression of 0.87 and expression increased during plant infection. Overall expression of effectors (0.57) and secreted (0.63) proteins was lower but increased 84 h after infection, indicating host colonisation‐related activity.

### 2.5 Species‐ and Lineage‐Specific Effector Repertoires in *C. lupini*

**FIGURE 7:**
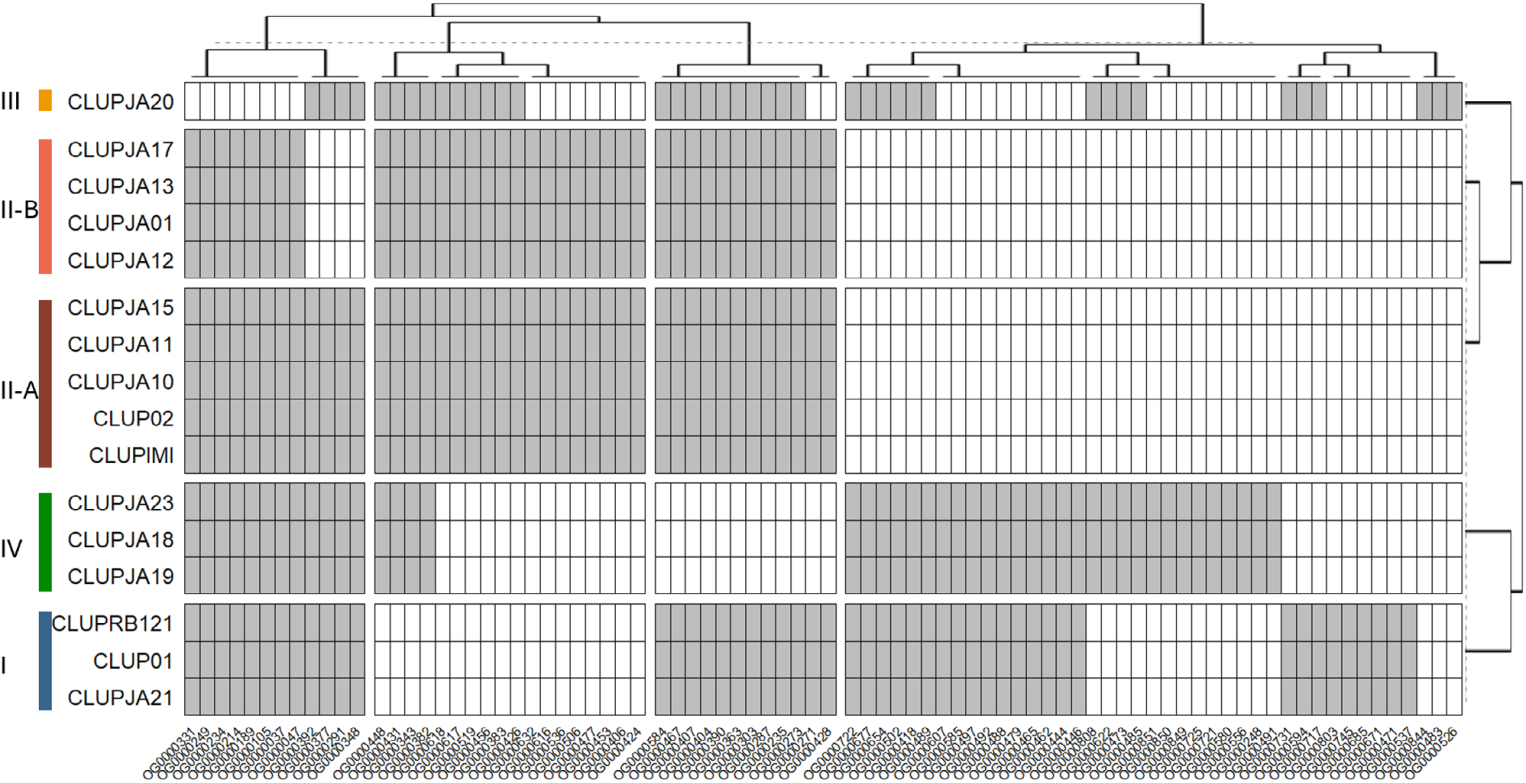
Lineage‐specific predicted effector‐containing orthogroups. See Table S3 for predicted gene functions. Grey is present, white is absent. Euclidian distance dendrograms are shown above and right of the plot.

Distinct virulence patterns for each strain were observed on white and Andean lupin ( *L. mutabilis* ; Alkemade et al. 2023; Alkemade et al. 2021). Virulence patterns on Andean lupin, however, did not correspond to assigned lineages, with great variation within and no difference between lineages for virulence on Andean lupin (Figure S3h–j). On white lupin, Lin I, III, and IV showed weak virulence, whereas virulence levels of Lin II‐A and B were high. Effectors play an important role in virulence and were upregulated upon infection (Figure 6g). To explain observed virulence patterns and to identify *C. lupini‐* and lineage‐specific effector‐containing orthologous genes, a genome‐wide association study (GWAS)‐like approach was used. Predicted orthologous genes of mature effector proteins were used as input, and species, lineage, and virulence data were used as “phenotype” input (Alkemade et al. 2021). This resulted in four effector orthogroups appearing to be unique for *C. lupini* (Table S3, Figure S13). These four orthogroups (OG0000520, −539, −540, and −542) contained XP_049136846.1, XP_049146329.1, XP_049145599.1, and XP_049136058.1 of the reference genome CLUP02. The predicted effector XP_049136846.1, encoding a peptidase A1 domain and upregulated upon plant infection (Table S3), only appears to match with effectors of *Seridium cardinale*, the cereal pathogen *Pyrenophora teres* and *Microdochium nivale* (Figure S14). XP_049146329.1 was only shared with closely related *C. tamarilloi*, *C. costaricense* , and *C. filicis* , whereas XP_049145599.1 and XP_049136058.1 yielded no matches to any other species. In Lin I, seven unique effector orthogroups were identified, nine in II, one in II‐A, one in II‐B, three in III, and 10 in IV (Figure 7, Table S3). From the nine Lin II unique orthogroups, four (OG0000453, −016, −632 and −506) contained CLUP02 genes that were upregulated in planta, encoding a LysM domain, a fungal lipase, a protein kinase and a EC51a protein, respectively. One Lin II‐specific orthogroup (OG0000536) contains secreted‐in‐xylem (SIX) proteins, which are commonly associated with pathogenicity. Two Lin I‐specific orthogroups (OG0000537 and −745) contain KAK1721939.1 and KAK1709830.1, which encode a necrosis‐inducing Ecp2 effector and a biotrophy‐associated secreted protein 3 with a Zn(2)‐C6 fungal‐type domain, respectively. Lin III‐specific OG0000526 contains a protein homologous to a CEC3 effector of *C. trifolium*. Based on virulence on white lupin (cultivar Feodora), only one orthogroup (OG0000033) could be identified, encoding a protein with a cellulose‐binding domain. No orthogroups were identified based on virulence on Andean lupin. Using the same approach, two Lin II‐ and one Lin IV‐specific CAZyme orthogroups were identified, corresponding to a glycoside hydrolase, calcium‐translocating P‐type ATPase, and chitinase, respectively (Table S3).

### 2.6 De‐Repression of LTR‐Copia Upon Infection

As TEs have been linked to virulence in various fungal plant pathogens, we compared TE content to *C. lupini* virulence. While no correlation was observed between total TE content and virulence on white lupin (*r* = 0.28, *p* = 0.3), significant correlations were identified for the specific TE superfamilies LTR‐Gypsy (*r* = 0.5, *p* = 0.05), LINE‐Tad1 (0.83, *p* = 7.5e−5) and DNA‐hAT (*r* = 0.69, *p* = 0.003; Figure S5). No correlation between TE content and virulence on Andean lupin was observed. Expression data showed that upon infection of white lupin, apart from a slight de‐repression of LTR‐Copia (*p* = 0.009), expression remained stable and there was no relaxation of TE repression (Figure 5e).

### 2.7 Accessory Chromosome Not Directly Involved in Virulence

ACs are often involved in virulence (Bertazzoni et al. 2018), so we had a closer look at chromosome 11. AC 11 is conserved amongst Lin II isolates, partly present in Lin III and IV, and absent in Lin I (Figure 4b). A total of 54 genes was annotated on AC 11 of CLUP02, with none predicted to encode for a secreted, effector or CAZyme protein. No metabolic gene clusters were identified either (Figure 3c). The gene‐poor AC 11 was clearly divided in gene‐ and TE‐rich regions (Figure 4b). Orthologues were shared amongst lineages II, III, and IV and most clade 1 species (CCOS01, CFIL01, CABS01, CLIM01, CCUS01, and CSCO01), indicating that those species probably contain an AC as well (Figure 4a). Most AC 11 genes encode uncharacterized proteins, but one gene encodes a zinc finger C2H2‐type (XP_049138510.1; Table S4). Two proteins, XP_049138504.1 and XP_049138485.1, were unique for Lin II, encoding an uncharacterized protein and a ubiquitin‐like protease family profile domain‐containing protein. The absence of secreted proteins, effectors, CAZymes, or metabolic gene clusters, the low expression upon plant infection (Figure 6f,g), the fact that Lin I did not show reduced virulence on Andean lupin, and that Lin I virulence on white lupin was not different compared to AC 11‐containing Lin III and IV (Figure S3), suggests that AC 11 is not directly involved in lupin colonisation.

## 3 Discussion

A global collection of *C. lupini* strains was sequenced to gain insights into the evolution and genetic variability of this asexual fungal plant pathogen. Based on conserved core genes, phylogenomics clearly showed that *C. lupini* was divided in four highly uniform lineages, matching the population analysis based on SNP profiling by Alkemade et al. (2023). The lack of genetic diversity, however, appeared at odds with the numerous differences in morphology and virulence observed within the species (Alkemade et al. 2021). Differences in TE content, gene content, whole‐genome alignment, and AC presence revealed that *C. lupini* is more diverse than previously detected, with Lin II clearly splitting into two distinct groups, II‐A and II‐B.

Pangenome analysis of *C. lupini* revealed significant genomic diversity, characterised by a stable core genome (80%) and dynamic accessory genome. This level of conservation is similar to three human fungal pathogens and *Saccharomyces cerevisiae* , where core gene estimates ranged between 80% and 90% (McCarthy and Fitzpatrick 2019), but is more conserved than the plant pathogens *Colletotrichum graminicola* (60%; Becerra et al. 2023), *F. oxysporum* f. sp. *cubense* (53%; van Westerhoven et al. 2024), and *Zymoseptoria tritici* (60%; Badet et al. 2020). While the core genes show limited copy number variation and high protein conservation, the accessory genome, including AC 11, exhibits great variability, with relaxed selective pressure and closer proximity to TEs. Genes found in all *C. lupini* strains but not in the rest of the CaSC also showed low conservation and relaxed selection pressure. This variability suggests that the accessory genome and *C. lupini* ‐specific genes play a key role in generating genetic diversity, as is seen for other *Colletotrichum* species (Lapalu et al. 2024; Liang et al. 2024) and asexual fungal plant pathogens (van Westerhoven et al. 2024; Langner et al. 2021). Pangenome analysis, however, greatly depends on the diversity of strains included in the analysis and should be interpreted carefully.

TEs contribute to intraspecific variation and play an important role in the evolution of fungi (Wells and Feschotte 2020; Grandaubert, Balesdent, and Rouxel 2014; Faino et al. 2016; Seidl and Thomma 2017). Especially in partially clonal fungal plant pathogens, such as *Magnaporthe*, *Fusarium*, and *Verticillium*, TEs have been shown to be crucial for host adaptation (Badet et al. 2020; Faino et al. 2016; Nakamoto, Joubert, and Krasileva 2023). In *Colletotrichum*, TE content was shown to be responsible for genome size variation and was linked to diversification (Tsushima et al. 2019; Gan et al. 2021). The most common TEs in *C. lupini* are the LTR retrotransposons Copia and Gypsy, which is similar to that found for other *Colletotrichum* species (Rao et al. 2018). In *C. lupini* , Copia elements especially, and Gypsy elements to a lesser extent, seem to have contributed most to genome expansion within the species. In the pathogens with clonal lineages *Rhynchosporium commune*, *Blumeria graminis*, and *Z*. *tritici*, Gypsy elements make up about half of the TE copies in the genome and have contributed to recent genome expansions and variability (Stalder et al. 2023; Frantzeskakis et al. 2018; Oggenfuss et al. 2021). This highlights that TE‐mediated variation can provide an important source for adaptation when meiosis is rare or lacking. Retrotransposons have been shown to be de‐repressed upon stress (Fouché et al. 2020; Torres, Thomma, and Seidl 2021; Gupta et al. 2023). In *C. lupini* Copia elements were the only TEs being de‐repressed upon infection. This has also been observed in *Z. tritici*, in which the other TE elements were only upregulated during the less stressful saprophytic stages of the pathogen's life cycle (Fouché et al. 2020). In *Botrytis cinerea*, Copia elements have even been directly linked to increasing virulence (Porquier et al. 2021). Besides de‐repression, correlations between white lupin virulence and LTR‐Gypsy, LINE‐Tad1, and DNA‐hAT TE content were observed. These TE types have been indicated to improve the adaptability of fungal pathogens (Muszewska et al. 2019). Besides the benefits, TE de‐repression increases the risk of TE insertions into essential genes and the rate of deleterious rearrangements (Fouché et al. 2022). To increase accuracy of transposon prediction, chromosome‐level assemblies could be used to provide a more complete understanding of TE biology in *C. lupini* .

The *Colletotrichum* genus is thought to consist of 10 core chromosomes and a variable number (0–8) of ACs (Wang et al. 2023). In *C. lupini* , a single AC has been identified that was TE dense and highly variable in size, which is common for ACs seen in other fungal species (Habig and Stukenbrock 2020; Bertazzoni et al. 2018). As this analysis has been based on alignments to a reference genome, long‐read assemblies will be required to confirm the presence of one or more ACs in *C. lupini* . In fungal plant pathogens, ACs are often associated with pathogenicity (Bertazzoni et al. 2018). In *F. oxysporum* f. sp. *lycopersici*, transferring AC 14 converted nonpathogenic strains into pathogens (Ma et al. 2010). In *Colletotrichum* species *C. asianum* (Wang et al. 2023), *C. higginsianum* (Dallery et al. 2017; Plaumann et al. 2018) and *C. lentis* (Bhadauria et al. 2019), ACs are effector‐rich and involved in mediating virulence. In *C. lupini* , however, AC 11 did not contain any effector, secreted or CAZyme‐encoding genes or any metabolic gene clusters. Although Lin I strains showed low virulence on white lupin, the absence of AC 11 did not hamper virulence on Andean lupin (Alkemade et al. 2021; Alkemade et al. 2023), indicating no direct function related to virulence. The lack of effectors on ACs was also shown for *C. graminicola* (Becerra et al. 2023), but deletion of its AC 12 still hindered full virulence (Ma et al. 2023). In *B. cinerea* and *Z. tritici*, no direct link to pathogenicity was found and ACs are suggested to be required for niche adaptation rather than pathogenicity (Van Kan et al. 2017; Habig, Quade, and Stukenbrock 2017). While virulence factors are a prominent feature of ACs in fungal plant pathogens, these chromosomes can serve diverse functions beyond pathogenicity, reflecting the dynamic nature of fungal genomes and their adaptation strategies in various ecological environments (Bertazzoni et al. 2018; Habig and Stukenbrock 2020). Performing competition experiments and infection trials on a broader spectrum of lupin species with strains with and without AC 11 might further elucidate its function.

Effectors are not randomly distributed across the *C. lupini* genome but are mostly localised in gene‐poor and TE‐rich regions. This has been observed for other fungal plant pathogens such as *C. higginsianum* (Tsushima et al. 2019) and *F. oxysporum* (van Westerhoven et al. 2024) and is considered common but not ubiquitous across fungi (Dong, Raffaele, and Kamoun 2015; Torres et al. 2020). This co‐localisation facilitates variability of effectors, which can contribute to niche adaptation. Effectors are often less conserved than core genes (Badet et al. 2020), but in *C. lupini* , pairwise identity amongst effectors was similar to core genes. This suggests that the presence/absence of effectors might be more common than amino acid substitutions to alter virulence and host specificity. In *F. oxysporum*, for example, the presence or absence of specific effectors greatly influences host range (van Westerhoven et al. 2024; Batson et al. 2021). Even though observed virulence on the tested Andean lupin varieties did not correspond to described lineages, presence/absence variation of predicted effectors did, indicating adaptive lineage‐specific effector repertoires. One of the four effectors unique to *C. lupini* was upregulated during host colonisation and encodes a peptidase A1 domain, which has been linked to virulence in various fungal pathogens (Qian et al. 2022; Krishnan et al. 2018). In Lin II, two lineage‐specific effectors were highly upregulated, one encoding a fungal lipase, shown to contribute to virulence in *F. graminearum* on wheat (Voigt, Schäfer, and Salomon 2005), and an EC51a protein homologous to a candidate effector in *C. higginsianum* (Gan et al. 2021). The gene function of accessory effectors identified in this study and its relevance for host specificity should be further characterised by knockouts or gene silencing.

In conclusion, we discovered considerable underlying genetic variability in *C. lupini* lineages that was not apparent from earlier surveys on sequence variation of SNPs. Our analyses do not rule out the possibility of rare sexual reproduction in *C. lupini* . Yet, the key components of diversity in chromosome structure, TE activity, and the presence or absence of putative effector genes that we detected can all arise through mechanisms independent of sexual reproduction. These findings now open up the potential to identify specific pathogenicity factors behind colonisation of lupins, disease symptoms, and crop losses. Understanding mechanisms that generate genetic variability in fungal plant pathogens, especially in genes that cause disease, is crucial for designing durable control and breeding strategies and is vital to sustainably reduce their impact on global food production.

## 4 Experimental Procedures

### 4.1 Culture Collection and DNA Extraction

A total of 16 *C. lupini* strains were collected from public culture collections and lupin plants with symptoms by collaborators worldwide, representing 10 countries across five continents (Table S1, Figure S1). All isolates were single‐spored and maintained on potato dextrose agar (PDA; Carl Roth) at 22°C in the dark as working cultures. Isolates were stored in 25% glycerol at −80°C for long‐term storage. To extract DNA, mycelium from single‐spore cultures was collected after 10 days of growing on PDA at 22°C with a sterile spreader after flooding the Petri dish with 2 mL of sterile double‐distilled water. Genomic DNA was isolated with a CTAB extraction protocol described in Minas et al. (2011).

### 4.2 Sequencing and Genome Assembly

Whole‐genome shotgun sequences were obtained through 150 bp paired‐end sequencing at a depth of  > 115× coverage on an Illumina NovaSeqX Plus platform by Genome Quebec (Quebec, Canada). Raw reads were trimmed for adapter sequences and filtered for a phred quality of 20 using FastP v. 0.23.4 (Chen et al. 2018). Quality was evaluated using FastQC v. 0.12.1 (Andrews 2010) and summarised using MultiQC v. 1.14 (Ewels et al. 2016). Reads were assembled using SPAdes v. 3.15.5 (‐‐isolate; Bankevich et al. 2012), and resulting scaffolds were filtered for minimum length (350 bp) and min (30×) and max (1000×) coverage. Assemblies were screened and cleaned from potential contamination using BLASTn (nt database) and BlobTools v. 1.1.1 (Laetsch and Blaxter 2017). QUAST v. 5.2.0 (Gurevich et al. 2013) was used to assess assembly quality and length, and BUSCO v. 5.4.7 (Manni et al. 2021) with the glomerellales_odb10 dataset was used to assess completeness. Kmers were counted using Jellyfish v. 2.3.0 (Marçais and Kingsford 2011). The resulting assemblies were compared to publicly available *C. lupini* and CaSC assemblies (Table S1). To identify core and accessory chromosomes, homology‐based scaffolding, with CLUP02 as a reference, was performed using RagTag v. 2.1.0 (Alonge et al. 2022).

### 4.3 Transposable Elements and Repeat Annotation

Repetitive elements were identified in the complete genomes of CLUP02 and CLUP01 using Repeatmodeler v. 2.0.4 (Flynn et al. 2020) with options “‐engine ncbi” and “‐LTRStruct”. A consensus library of the predicted repetitive sequences and previously described *Colletotrichum* transposons (Dallery et al. 2017) was created, filtering for identical sequences (identity and coverage  > 80%). Repeats were classified using the Repeatclassifier of RepeatMasker v. 4.1.5 (Flynn et al. 2020) and the created consensus library was split into known and unknown repetitive elements. Genomes were annotated for TEs and masked in four steps using RepeatMasker with a cut‐off value of 250. First, simple repeats were identified and soft‐masked based on fungal repeats present in a combined Rebase (Bao, Kojima, and Kohany 2015; release 20181026) and Dfam v. 3.7 (Storer et al. 2021) database, followed by the identification and hard masking of complex repeats based on the *Glomerella* repeats present in the database and known and unknown repeats of the consensus library, ignoring short ( < 100 bp) repeats. Masked short‐read assemblies were used for gene annotations, whereas RagTag assemblies were used to determine the TE landscape.

### 4.4 SNP and TE Insertion Analysis

SNPs were called by mapping reads to reference genome CLUP02 using BWA v. 0.7.17‐r1188 (Li 2013). Resulting SAM files were converted to BAM files that were indexed and sorted using SAMtools v. 1.17 (Danecek et al. 2021). Variant calling was performed using mpileup of BCFtools v. 1.14 and variants were filtered for quality (Q20), minimum sequencing depth (2), mean sequencing depth (5), minor allele count (2), minor allele frequency (0.01), and missing data (0.95) using VCFtools v. 0.1.16 (Auton & Marcketta, 2009). TE insertions were called using ngs‐te‐mapper2 (Linheiro and Bergman 2012). Sequencing reads were mapped against the consensus TE library, using a window of 100 bp, to identify reference and non‐reference TEs. A PCA was created from whole‐genome SNPs and reference TE insertions using the *prcomp* function in R v. 4.3.1 (R Core Team 2020). Selective sweep analysis was performed using SweeD v. 4.0.0 (Pavlidis et al. 2013) with a 10 kb sliding window using the above‐mentioned SNP dataset. The analysis was performed for all isolates together and for each lineage (I, II, and IV) separately. Results are expressed as composite‐likelihood ratio (CLR).

### 4.5 Gene Prediction and Functional Annotation

Gene annotation was performed on masked genomes with four rounds of the MAKER v. 3.01.04 annotation pipeline. In the first round, transcript sequences of CLUP02 were used as EST evidence, and CSCO01 and CFIO01 transcripts were used as alternative EST evidence, protein sequences of CLUP02, CLUP01, CPAR01, CSCO01, and CFIO01 were used for protein homology evidence (Table S1). The following three rounds were performed with a CLUP02‐trained version of AUGUSTUS v. 3.5.0 (Keller et al. 2011) and SNAP v. 2006‐07‐28 (Korf 2004), which was trained each consecutive annotation round. Predicted proteomes were assessed for completeness using BUSCO (glomerellales_odb10).

The secretome was defined by proteins with a signal peptide but no transmembrane domain as predicted by Phobius v. 1.01 (Käll, Krogh, and Sonnhammer 2004), TMHMM v. 2.0 (Krogh et al. 2001), WoLF PSORT (Horton et al. 2007), and SignalP v. 6.0 (Teufel et al. 2022) integrated within the EffHunter v. 1.0 (Carreón‐Anguiano et al. 2020) pipeline with a protein length range of 0–13,000 amino acids and a minimum of 0 cysteine residues. Predicted secretome was screened for effector candidates by EffectorP v. 3.0 (Sperschneider and Dodds 2022). Protein functions were predicted using Interproscan v. 5.63–95.0 (Jones et al. 2014), adding ‐goterms ‐iprlookup and ‐pathway information. Carbohydrate‐active enzymes (CAZymes) were identified using the dbCAN3 server with the HMMER:dbcan, HMMER:dbcan‐sub, and DIAMOND:CAZy tools (Buchfink, Xie, and Huson 2015; Finn, Clements, and Eddy 2011; Zheng et al. 2023). Proteins were classified as a CAZyme if predicted by each of the three tools. Secondary metabolite gene clusters were predicted using the online fungal version of antiSMASH v. 7.0 (Blin et al. 2023).

### 4.6 Comparative Genomics and Pangenome Analysis

Whole‐genome alignments were performed using nucmer (options ‐‐maxmatch ‐c 80) in MUMmer v. 3.1 (Kurtz et al. 2004). Alignment was performed against the repeat‐masked reference genome of CLUP02. Resulting files were filtered for 1 to 1 alignments with a sequence identity of  > 80% and an alignment length of  > 500 bp. Alignment plots were created using Circos (Krzywinski et al. 2009) and R package *circlize* (Gu et al. 2014). Phylogeny and orthologous groups (OGs) were identified using OrthoFinder v. 2.5.4 (Emms and Kelly 2019) with the “‐M msa” option on predicted protein‐coding genes of *C. lupini* and CaSC species. OGs present in all isolates were considered core CaSC, and genes only present in *C. lupini* were considered *C. lupini* ‐specific. Within *C. lupini* , OGs present in all isolates were considered core and genes present in fewer isolates were considered accessory. Genes were further divided into the following categories: CAZyme, secreted, effector and Chr11 genes. Percent identity of multiple protein sequence alignments was determined for each OG using MAFFT v. 7.505 (Katoh and Standley 2013). The number of nonsynonymous and synonymous substitutions (dN/dS) per OG was determined by using the aligned proteins, followed by a codon‐guided nucleotide alignment using PAL2NAL v. 14.1 (Suyama, Torrents, and Bork 2006). This codon‐guided alignment was used together with OG gene trees generated by Orthofinder to infer dN/dS values using CODEML from PAML v. 4.9 (Yang 2007). For each category, intersection, or distance to a selective sweep ( > CLR 10) or TE was determined with the “closest” and “intersect” option of BEDTools v. 2.30 (Quinlan and Hall 2010). Gene distribution along the genome for each gene category was assessed by coefficients of variation (CV), and it was tested if genes followed a Poisson distribution. A fictional dataset of randomised genes was used as control. To identify *C. lupini‐* and lineage‐specific effectors, a GWAS‐like approach, using *statgenGWAS* (van Rossum et al. 2020) and Orthofinder generated gene‐count matrices, was performed. Identified genes were manually checked for specificity using BLAST.

### 4.7 RNA‐Seq Analysis

Expression data of *C. lupini* growing for 24 h in liquid medium (Czapek‐Dox 0.5 g/L) and during white lupin infection 24 and 84 h after inoculation (Dubrulle et al. 2020) was downloaded from NCBI (Table S5). Raw reads were trimmed using Trimmomatic v. 0.39 (Bolger, Lohse, and Usadel 2014), with phred score of 33, a minimum length of 50, and a sliding window of 5:10. Quality was assessed using FastQC. Reads were mapped to the reference genome (CLUP02), using STAR v. 2.7.10b (Dobin et al. 2013), allowing for multiple read mapping (parameters set as ‐‐outFilterMultimapNmax 100 ‐‐winAnchorMultimapNmax 200 ‐‐outFilterMismatchNmax 3). HTSeq‐count v. 2.0.5 (Anders, Pyl, and Huber 2015) was used to retrieve counts per gene and TE. Counts were normalised, and DEGs were identified using the R package *DESeq2* (Love, Huber, and Anders 2014) by analysing all treatments together.

### 4.8 Data Analysis

Statistical analyses were performed with R using the packages *stats* and *multcomp* (Hothorn, Bretz, and Westfal 2012). For genome characteristics that did not follow assumptions of normality of residuals and homogeneity of variance, square root or logit (percentage data) were transformed. A Tukey HSD test (*p* ≤ 0.05) was applied for pairwise mean comparisons. Differences between gene categories were compared using a Bonferroni‐adjusted Dunn's test. Expression data was analysed through mean comparison to the control (24 h in liquid medium) using a Kruskal–Wallis test because a normal distribution of the residuals could not be achieved.

## Conflicts of Interest

The authors declare no conflicts of interest.

## Supporting information


**Figure S1.** Global lupin production and distribution of *Colletotrichum lupini*. Lupin production of 2020 in tonnes, sources are: FAOSTAT (2021), Gulisano et al. (2019), and Akale et al. (2019). Circles in blue indicate *C. lupini* lineage I, red is II, orange is III, and green is IV. Asterisks indicate isolates collected before 1990. Figure is based on figure shown in Alkemade et al. (2023).

**Figure S2.** Differences in (a) genome size, (b) GC content (%), (c) gene content, and (d) effector content between species belonging to clade 2, 3, 4, and 5 of the *Colletotrichum acutatum* species complex (CaSC), clade 1 of the CaSC, and *C. lupini* . Uppercase letters within plots indicate significant differences between groups (Tukey HSD, *p* < 0.05).

**Figure S3.** Variation in (a) genome size, (b) gene content, (c) effector content, (d) GC content (%), (e) transposable element (TE) content (%), (f) unmapped contigs to reference genome CLUP02, (h) virulence on white lupin ( *Lupinus albus* ) cultivar Feodora, (i) virulence on Andean lupin ( *L. mutabilis* ) Lup‐17, and (j) virulence on Andean lupin Lup‐100, between different *Colletotrichum lupini* lineages. Uppercase letters within plots indicate significant differences between strains (Tukey HSD, *p* < 0.05). Lineage III is not included in statistical analysis as only one sample was available. Virulence data was collected from Alkemade et al. (2023).

**Figure S4.** Whole‐genome alignments between CLUP02 (Lin II) and CLUP01 (Lin I). The outer bands indicate chromosomes of CLUP02 (red) and CLUP01 (blue). Syntenic regions (80% identity, > 500 bp) are linked with different coloured ribbons corresponding to a chromosome from CLUP02.

**Figure S5.** Correlation between chromosome size (Mb) and total transposable element (TE) content (%), (a) chr1, (b) chr2, (c) chr3, (d) chr4, (e) chr5, (f) chr6, (g) chr7, (h) chr8, (i) chr9, and (j) chr 10. Correlation between genome size (Mb) and total content (Mb) of transposon subfamily, (k) LTR‐Copia, (l) LTR‐Gypsy, (m) unknown repeats, (n) DNA‐hAT, (o) DNA‐IS3EU, (p) DNA‐MULE‐MuDR, and (q) LINE‐Tad1. Correlation between virulence on white lupin expressed as standardise area under the disease progress curve (sAUDPC) and total content (Mb) of transposon subfamily, (r) LTR‐Gypsy, (s) LINE‐Tad1, and (t) DNA‐hAT.

**Figure S6.** Presence frequency of specific transposable element (TE) insertions across the 16 *Colletotrichum lupini* genomes.

**Figure S7.** Proportion of core orthogroups (present in all isolates) and accessory orthogroups (present ≥ 2 isolates but not all) across the proteome, carbohydrate‐active enzymes (CAZymes), secretome, secreted CAZymes and effectors.

**Figure S8.** Gene copy number variation in (a) core, (b) accessory, (c) carbohydrate‐active enzymes (CAZymes), and (d) effector orthogroups across the 16 *Colletotrichum lupini* genomes. In (a), AF = Africa, AU = Australia, EU = Europe, NA = North America, SA = South America. In (b–d) colours above plots indicate *C. lupini* lineages.

**Figure S9.** Total count and type of (a) secondary metabolite gene clusters and (b) carbohydrate‐active enzymes (CAZymes). CAZyme types are divided into glycoside hydrolase (GH), glycosyl transferase (GT), auxiliary activity (AA), carbohydrate esterase (CE), carbohydrate‐binding modules (CBM), and polysaccharide lyase activity (PL) categories.

**Figure S10.** Gene distribution per gene category. CV indicates coefficient of variation and * indicates that distribution is significantly different form a Poisson distribution (*p* < 0.05).

**Figure S11.** Proportion of genes per pangenome category closer than 10 kb to a transposable element (TE).

**Figure S12.** Selective sweep analysis and transposable element (TE) distribution across *Colletotrichum lupini* genome. (a) Genomic scans for selective sweeps for all 16 *C. lupini* isolates. CLR indicates composite‐likelihood‐ratio, and dashed line indicates CLR of 10, (b) analysis for lineage I isolates (*n* = 3), (c) analysis for lineage II isolates (*n* = 9) and (d) analysis for lineage IV isolates (*n* = 3). (e) Distribution of TEs across CLUP02 genome.

**Figure S13.** Predicted effector clusters of species within the *Colletotrichum acutatum* species complex that are specific to or absent from all or some *C. lupini* lineages. Euclidian distance dendrograms are shown above and right of the plot.

**Figure S14.** Maximum‐likelihood tree of OG0000520. Bootstrap support values ( > 95) are given at each node.

**Table S1.** Strain and genome details.

**Table S2.** Pangenome construction information.

**Table S3.** Functional information of predicted species/lineage specific effectors.

**Table S4.** Functional information of predicted genes on accessory chromosome 11.

**Table S5.** SRA accessions of used RNA‐seq data.

## Data Availability

The data that support the findings of this study are openly available in the Sequence Read Archive at https://www.ncbi.nlm.nih.gov/sra. The data sequenced in this study are available under BioProject PRJNA1099210. Accession, BioSample, and assembly identifiers are listed in Table S1.
