# Supplementary material for: Comparative Genomics Reveals Sources of Genetic Variability in the Asexual Fungal Plant Pathogen Colletotrichum lupini
Source: Mol Plant Pathol. 2024 Dec 13;25(12):e70039. doi: 10.1111/mpp.70039 (PMC11645255; doi:10.1111/mpp.70039)
Supplement: Supplementary file 1 — Figure S1. Global lupin production and distribution of Colletotrichum lupini. Lupin production of 2020 in tonnes, sources are: FAOSTAT (2021), Gulisano et al. (2019), and Akale et al. (2019). Circles in blue indicate C. lupini lineage I, red is II, orange is III, and green is IV. Asterisks indicate isolates collected before 1990. Figure is based on figure shown in Alkemade et al. (2023). [file MPP-25-e70039-s014.docx]

**
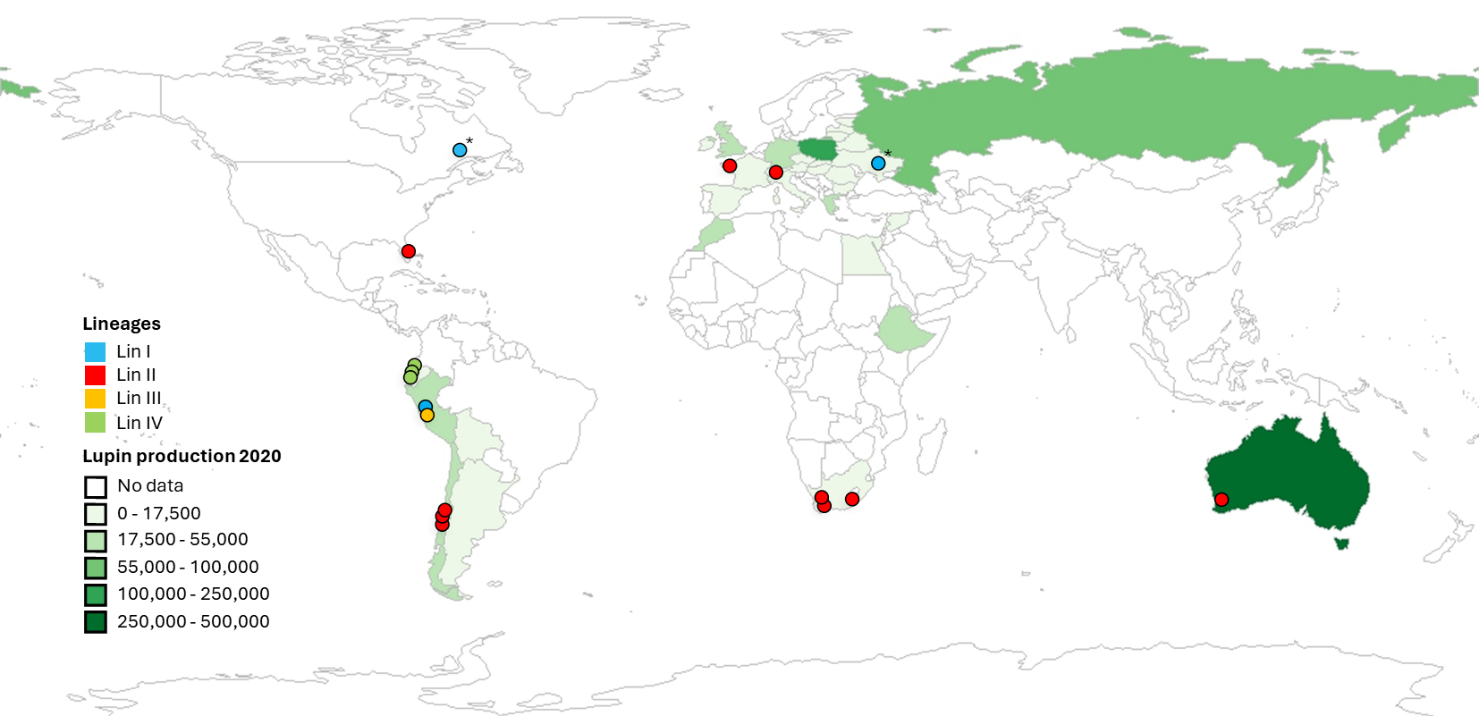
**

**Figure S1:** Global lupin production and distribution of *Colletotrichum lupini*. Lupin production of 2018 in tonnes, sources are: FAOSTAT (2021); Gulisano et al. (2019); Akale et al. (2019). Circles in blue indicate *C. lupini* lineage I, red is II, orange is III and green is IV. Asterisks indicate isolates collected before 1990. Figure is based on figure shown in Alkemade et al. (2023).

Akale, A. H., Alemu, M. W. and Asmamaw, M. B. (2019) Analyze production, utilization and its future trends of lupin in Ethiopia. *Am. J. Plant Sci.,* **10,** 1797-1812. <https://doi.org/10.4236/ajps.2019.1010127>

Alkemade, J. A., Baroncelli, R., Messmer, M. M. and Hohmann, P. (2023) Attack of the clones: Population genetics reveals clonality of *Colletotrichum lupini*, the causal agent of lupin anthracnose. *Mol. Plant Pathol.* <https://doi.org/10.1111/mpp.13332>

FAOSTAT (2021) Statistics Division of the Food and Agriculture Organization of the United Nations. <http://www.fao.org/faostat/en/#data/QC>

Gulisano, A., Alves, S., Neves Martins, J. and Trindade, L. M. (2019) Genetics and breeding of *Lupinus mutabilis*: an emerging protein crop. *Front. Plant Sci.,* **10,** 1385. <https://doi.org/10.3389/fpls.2019.01385>
