# Supplementary material for: Comparative Genomics Reveals Sources of Genetic Variability in the Asexual Fungal Plant Pathogen Colletotrichum lupini
Source: Mol Plant Pathol. 2024 Dec 13;25(12):e70039. doi: 10.1111/mpp.70039 (PMC11645255; doi:10.1111/mpp.70039)
Supplement: Supplementary file 2 — Figure S2. Differences in (a) genome size, (b) GC content (%), (c) gene content, and (d) effector content between species belonging to clade 2, 3, 4, and 5 of the Colletotrichum acutatum species complex (CaSC), clade 1 of the CaSC, and C. lupini . Uppercase letters within plots indicate significant differences between groups (Tukey HSD, p < 0.05). [file MPP-25-e70039-s004.docx]

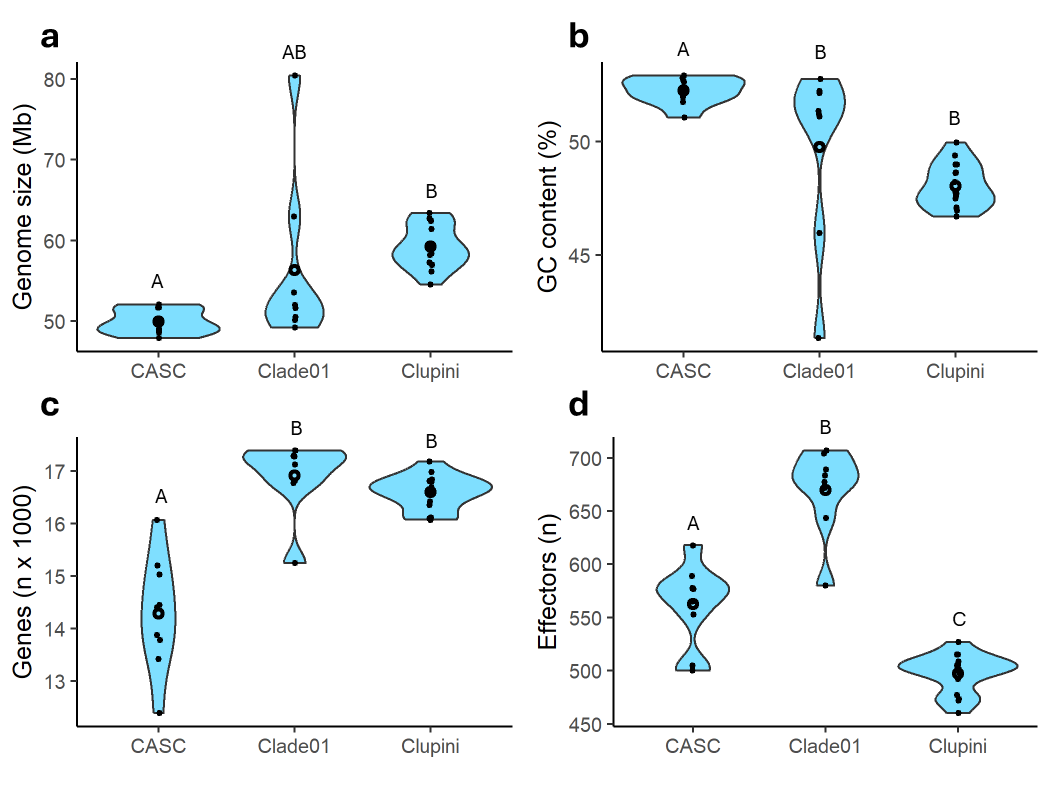


**Figure S2:** Differences in **(a)** genome size, **(b)** GC content (%), **(c)** Gene content and **(d)** effector content between species belonging to clade 2, 3, 4 and 5 of the *Colletotrichum acutatum* species complex (CaSC), clade 1 of the CaSC and *C. lupini*. Capital letters within plots indicate significant differences between groups (Tukey-HSD, p < 0.05).
