# Supplementary material for: Comparative Genomics Reveals Sources of Genetic Variability in the Asexual Fungal Plant Pathogen Colletotrichum lupini
Source: Mol Plant Pathol. 2024 Dec 13;25(12):e70039. doi: 10.1111/mpp.70039 (PMC11645255; doi:10.1111/mpp.70039)
Supplement: Supplementary file 3 — Figure S3. Variation in (a) genome size, (b) gene content, (c) effector content, (d) GC content (%), (e) transposable element (TE) content (%), (f) unmapped contigs to reference genome CLUP02, (h) virulence on white lupin ( Lupinus albus ) cultivar Feodora, (i) virulence on Andean lupin ( L. mutabilis ) Lup‐17, and (j) virulence on Andean lupin Lup‐100, between different Colletotrichum lupini lineages. Uppercase letters within plots indicate significant differences between strains (Tukey HSD, p < 0.05). Lineage III is not included in statistical analysis as only one sample was available. Virulence data was collected from Alkemade et al. (2023). [file MPP-25-e70039-s003.docx]

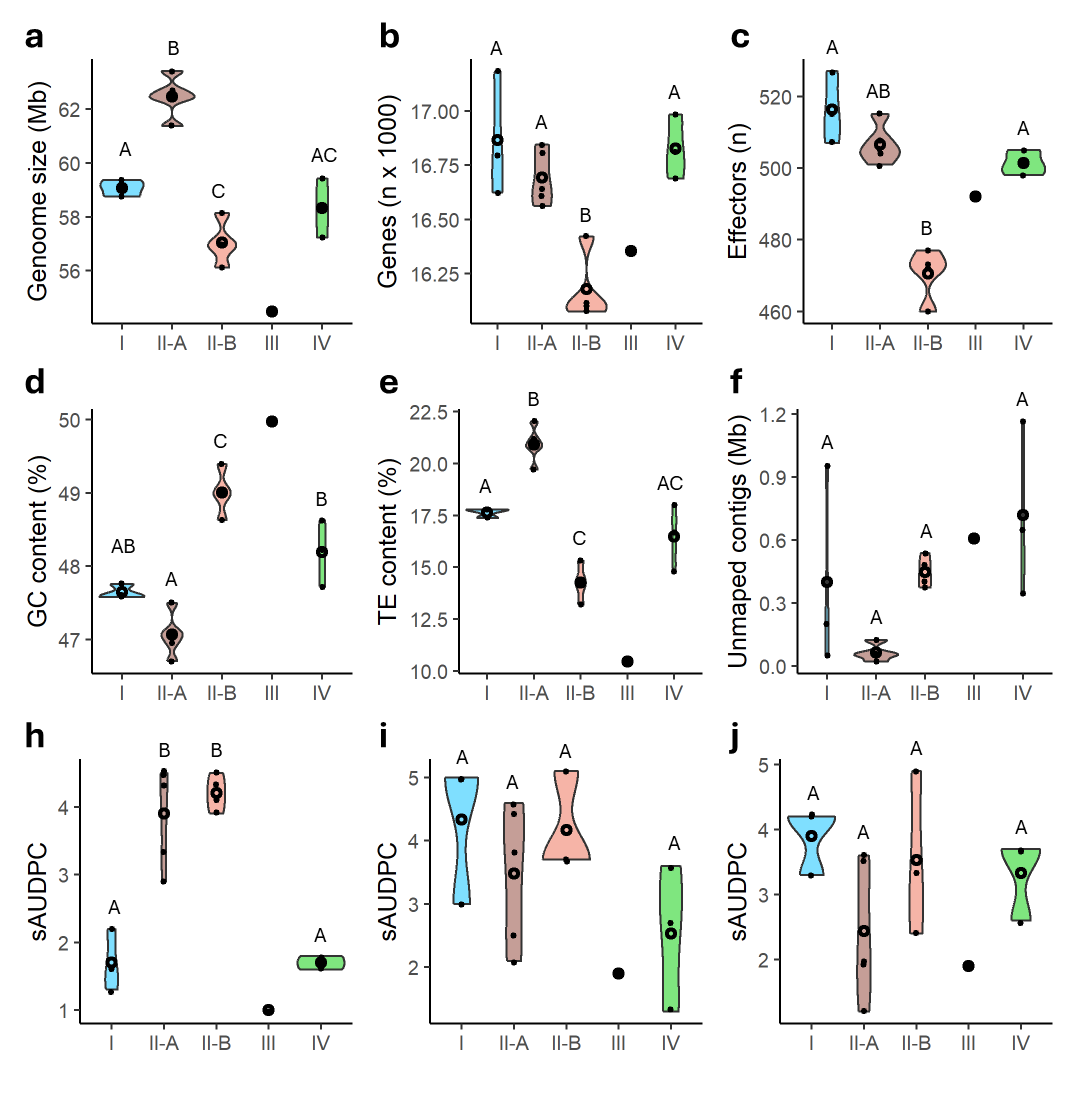


**Figure S3:** Variation in **(a)** genome size, **(b)** gene content, **(c)** effector content, **(d)** GC content (%), **(e)** TE content (%), **(f)** unmapped contigs to reference genome CLUP02, **(h)** virulence on white lupin (*Lupinus albus*) cultivar Feodora **(i)** virulence on Andean lupin (*L. mutabilis*) Lup-17 and **(j)** virulence on Andean lupin Lup-100, between different *Colletotrichum lupini* lineages. Capital letters within plots indicate significant differences between strains (Tukey-HSD, p < 0.05). Lineage III is not included in statistical analysis as only one sample was available. Virulence data was collected from Alkemade et al (2023).

Alkemade, J. A., Baroncelli, R., Messmer, M. M. and Hohmann, P. (2023) Attack of the clones: Population genetics reveals clonality of *Colletotrichum lupini*, the causal agent of lupin anthracnose. *Mol. Plant Pathol.* https://doi.org/10.1111/mpp.13332
