# Supplementary material for: Comparative Genomics Reveals Sources of Genetic Variability in the Asexual Fungal Plant Pathogen Colletotrichum lupini
Source: Mol Plant Pathol. 2024 Dec 13;25(12):e70039. doi: 10.1111/mpp.70039 (PMC11645255; doi:10.1111/mpp.70039)
Supplement: Supplementary file 4 — Figure S4. Whole‐genome alignments between CLUP02 (Lin II) and CLUP01 (Lin I). The outer bands indicate chromosomes of CLUP02 (red) and CLUP01 (blue). Syntenic regions (80% identity, > 500 bp) are linked with different coloured ribbons corresponding to a chromosome from CLUP02. [file MPP-25-e70039-s010.docx]

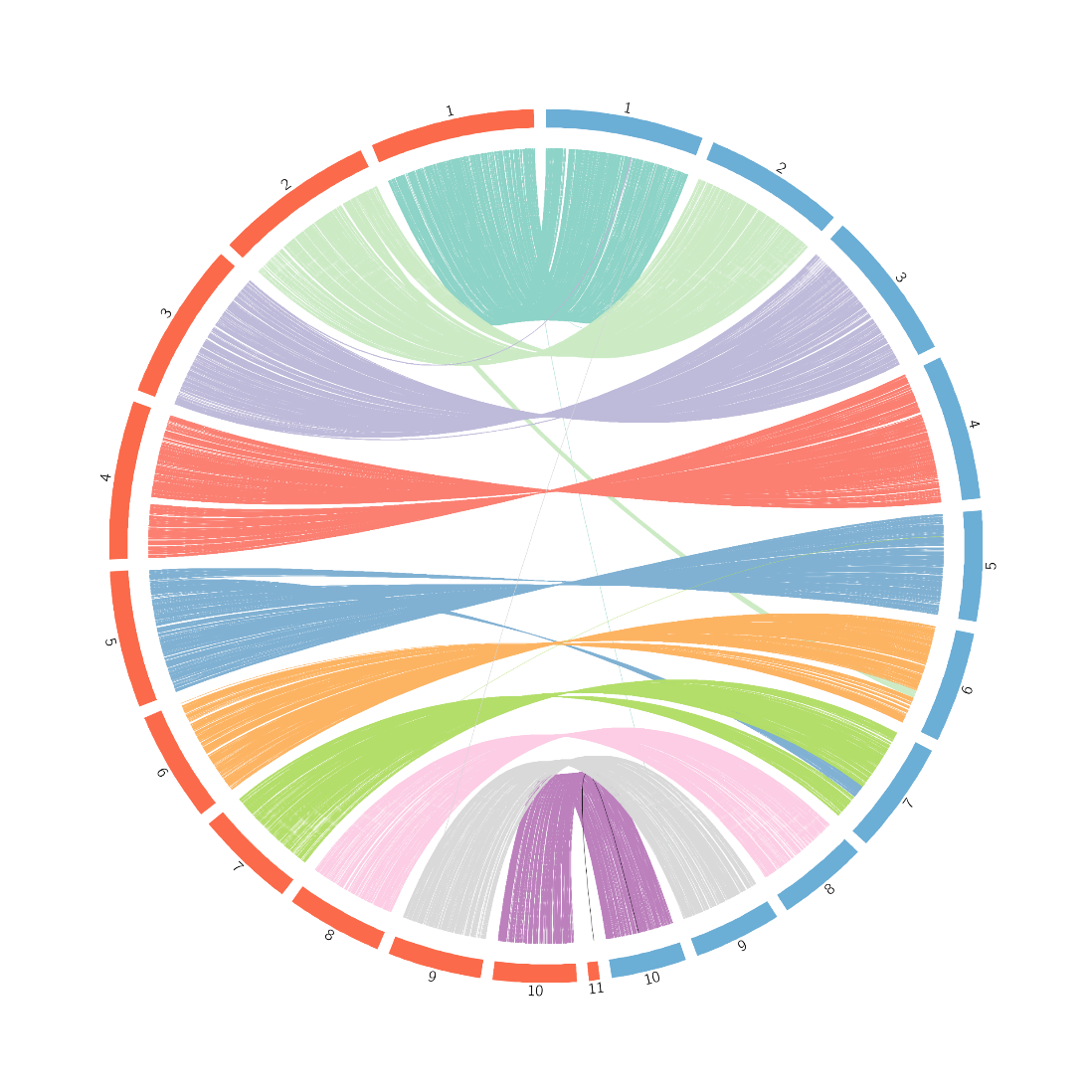


**Figure S4:** Whole-genome alignments between CLUP02 (Lin II) and CLUP01 (Lin I). The outer bands indicate chromosomes of CLUP02 (red) and CLUP01 (blue). Syntenic regions (80% identity, >500 bp) are linked with different colored ribbons corresponding to a chromosome from CLUP02.
