# Supplementary material for: Comparative Genomics Reveals Sources of Genetic Variability in the Asexual Fungal Plant Pathogen Colletotrichum lupini
Source: Mol Plant Pathol. 2024 Dec 13;25(12):e70039. doi: 10.1111/mpp.70039 (PMC11645255; doi:10.1111/mpp.70039)
Supplement: Supplementary file 5 — Figure S5. Correlation between chromosome size (Mb) and total transposable element (TE) content (%), (a) chr1, (b) chr2, (c) chr3, (d) chr4, (e) chr5, (f) chr6, (g) chr7, (h) chr8, (i) chr9, and (j) chr 10. Correlation between genome size (Mb) and total content (Mb) of transposon subfamily, (k) LTR‐Copia, (l) LTR‐Gypsy, (m) unknown repeats, (n) DNA‐hAT, (o) DNA‐IS3EU, (p) DNA‐MULE‐MuDR, and (q) LINE‐Tad1. Correlation between virulence on white lupin expressed as standardise area under the disease progress curve (sAUDPC) and total content (Mb) of transposon subfamily, (r) LTR‐Gypsy, (s) LINE‐Tad1, and (t) DNA‐hAT. [file MPP-25-e70039-s013.docx]

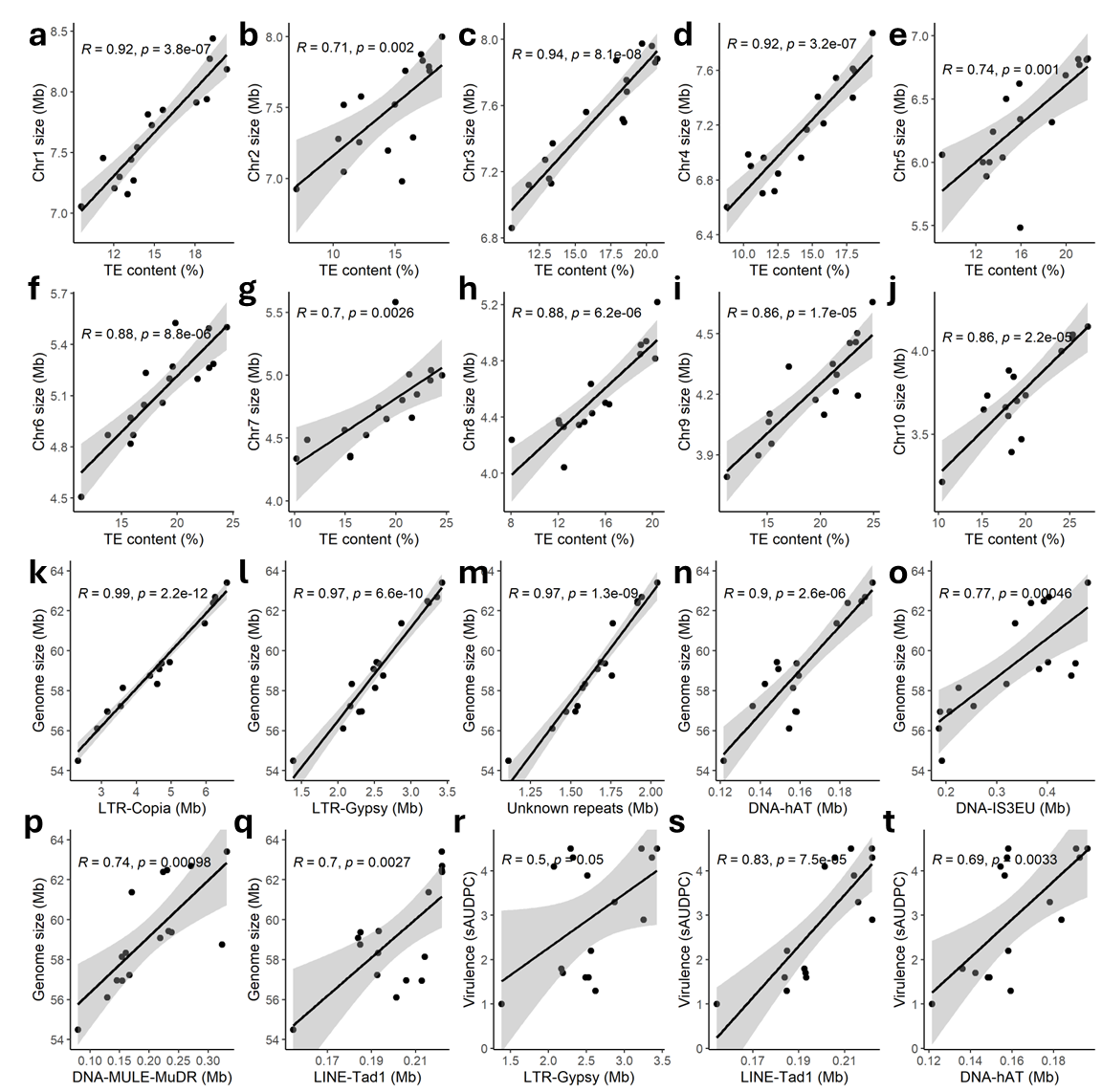


**Figure S5:** Correlation between chromosome size (Mb) and total TE content (%), **(a)** chr1, **(b)** chr2, **(c)** chr3, **(d)** chr4, **(e)** chr5, **(f)** chr6, **(g)** chr7, **(h)** chr8, **(i)** chr9 and **(j)** chr 10. Correlation between genome size (Mb) and total content (Mb) of transposon subfamily, **(k)** LTR-Copia, **(l)** LTR-Gypsy, **(m)** unknown repeats, **(n)** DNA-hAT, **(o)** DNA-IS3EU, **(p)** DNA-MULE-MuDR and **(q)** LINE-Tad1. Correlation between virulence on white lupin expressed as standardize area under the disease progress curve (sAUDPC) and total content (Mb) of transposon subfamily, **(r)** LTR-Gypsy, **(s)** LINE-Tad1, **(t)** DNA-hAT.
