# Supplementary material for: Comparative Genomics Reveals Sources of Genetic Variability in the Asexual Fungal Plant Pathogen Colletotrichum lupini
Source: Mol Plant Pathol. 2024 Dec 13;25(12):e70039. doi: 10.1111/mpp.70039 (PMC11645255; doi:10.1111/mpp.70039)
Supplement: Supplementary file 6 — Figure S6. Presence frequency of specific transposable element (TE) insertions across the 16 Colletotrichum lupini genomes. [file MPP-25-e70039-s005.docx]

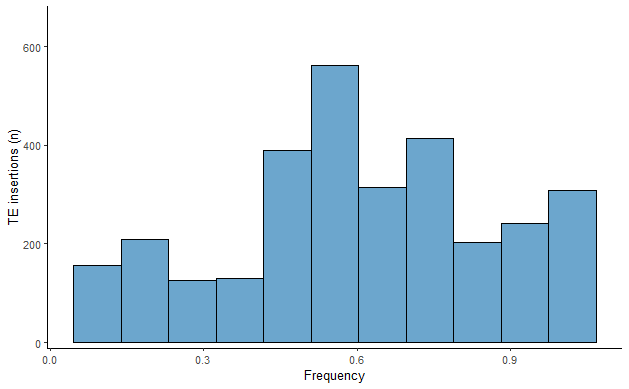


**Figure S6:** Presence frequency of specific TE insertions across the 16 *Colletotrichum lupini* genomes.
