# Supplementary material for: Comparative Genomics Reveals Sources of Genetic Variability in the Asexual Fungal Plant Pathogen Colletotrichum lupini
Source: Mol Plant Pathol. 2024 Dec 13;25(12):e70039. doi: 10.1111/mpp.70039 (PMC11645255; doi:10.1111/mpp.70039)
Supplement: Supplementary file 7 — Figure S7. Proportion of core orthogroups (present in all isolates) and accessory orthogroups (present ≥ 2 isolates but not all) across the proteome, carbohydrate‐active enzymes (CAZymes), secretome, secreted CAZymes and effectors. [file MPP-25-e70039-s019.docx]

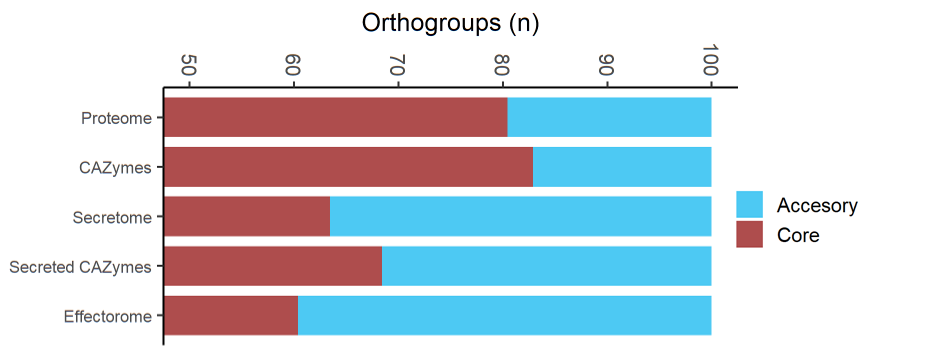


**Figure S7:** Proportion of core orthogroups (present in all isolates) and accessory orthogroups (present ≥ 2 isolates but not all) across the proteome, carbohydrate-active enzymes (CAZymes), secretome, secreted CAZymes and effectors.
