# Supplementary material for: Comparative Genomics Reveals Sources of Genetic Variability in the Asexual Fungal Plant Pathogen Colletotrichum lupini
Source: Mol Plant Pathol. 2024 Dec 13;25(12):e70039. doi: 10.1111/mpp.70039 (PMC11645255; doi:10.1111/mpp.70039)
Supplement: Supplementary file 8 — Figure S8. Gene copy number variation in (a) core, (b) accessory, (c) carbohydrate‐active enzymes (CAZymes), and (d) effector orthogroups across the 16 Colletotrichum lupini genomes. In (a), AF = Africa, AU = Australia, EU = Europe, NA = North America, SA = South America. In (b–d) colours above plots indicate C. lupini lineages. [file MPP-25-e70039-s007.docx]

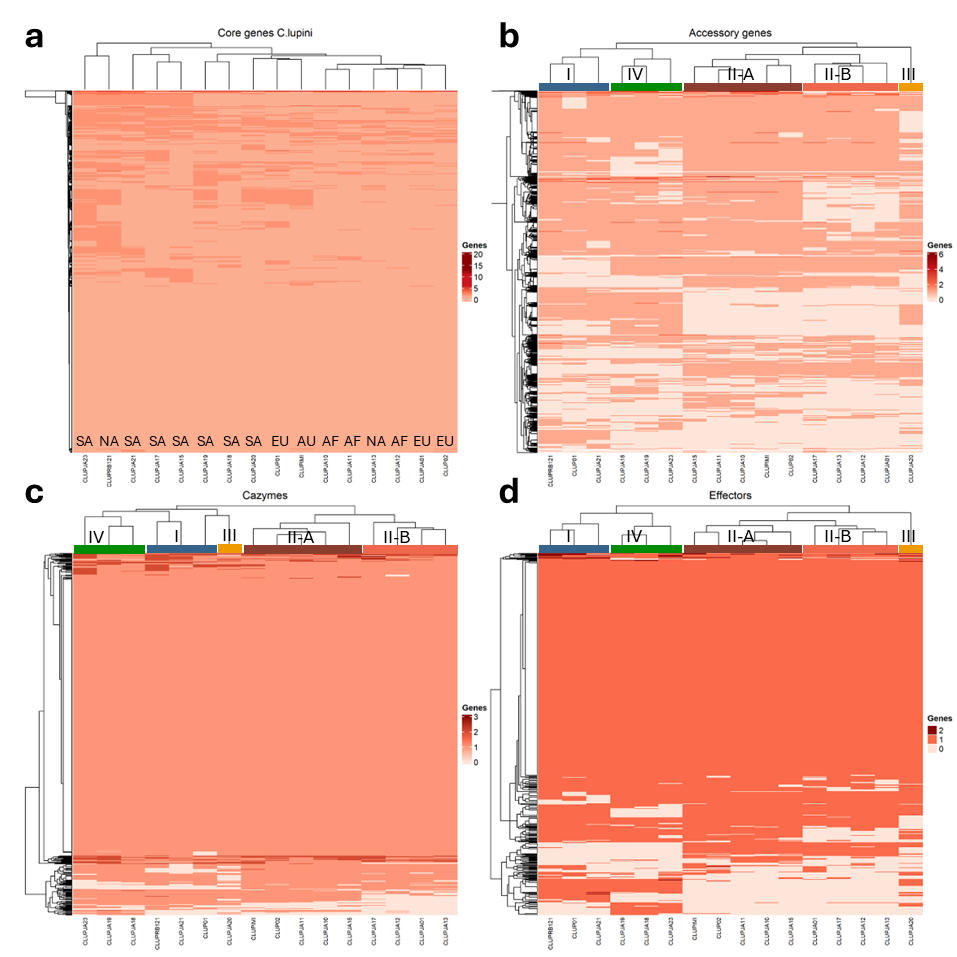
**Figure S8:** Gene copy number variation in **(a)** core, **(b)** accessory, **(c)** carbohydrate-active enzymes (CAZymes) and **(d)** effector orthogroups across the 16 *Colletotrichum lupini* genomes. In **a**, SA = South America, NA = North America, EU = Europe, AF = Africa and AU = Australia. In **b**, **c** and **d**, colors above plots indicate *C. lupini* lineages. Euclidian distance dendrograms are shown above and right of the plot.
