# Supplementary material for: Comparative Genomics Reveals Sources of Genetic Variability in the Asexual Fungal Plant Pathogen Colletotrichum lupini
Source: Mol Plant Pathol. 2024 Dec 13;25(12):e70039. doi: 10.1111/mpp.70039 (PMC11645255; doi:10.1111/mpp.70039)
Supplement: Supplementary file 9 — Figure S9. Total count and type of (a) secondary metabolite gene clusters and (b) carbohydrate‐active enzymes (CAZymes). CAZyme types are divided into glycoside hydrolase (GH), glycosyl transferase (GT), auxiliary activity (AA), carbohydrate esterase (CE), carbohydrate‐binding modules (CBM), and polysaccharide lyase activity (PL) categories. [file MPP-25-e70039-s017.docx]

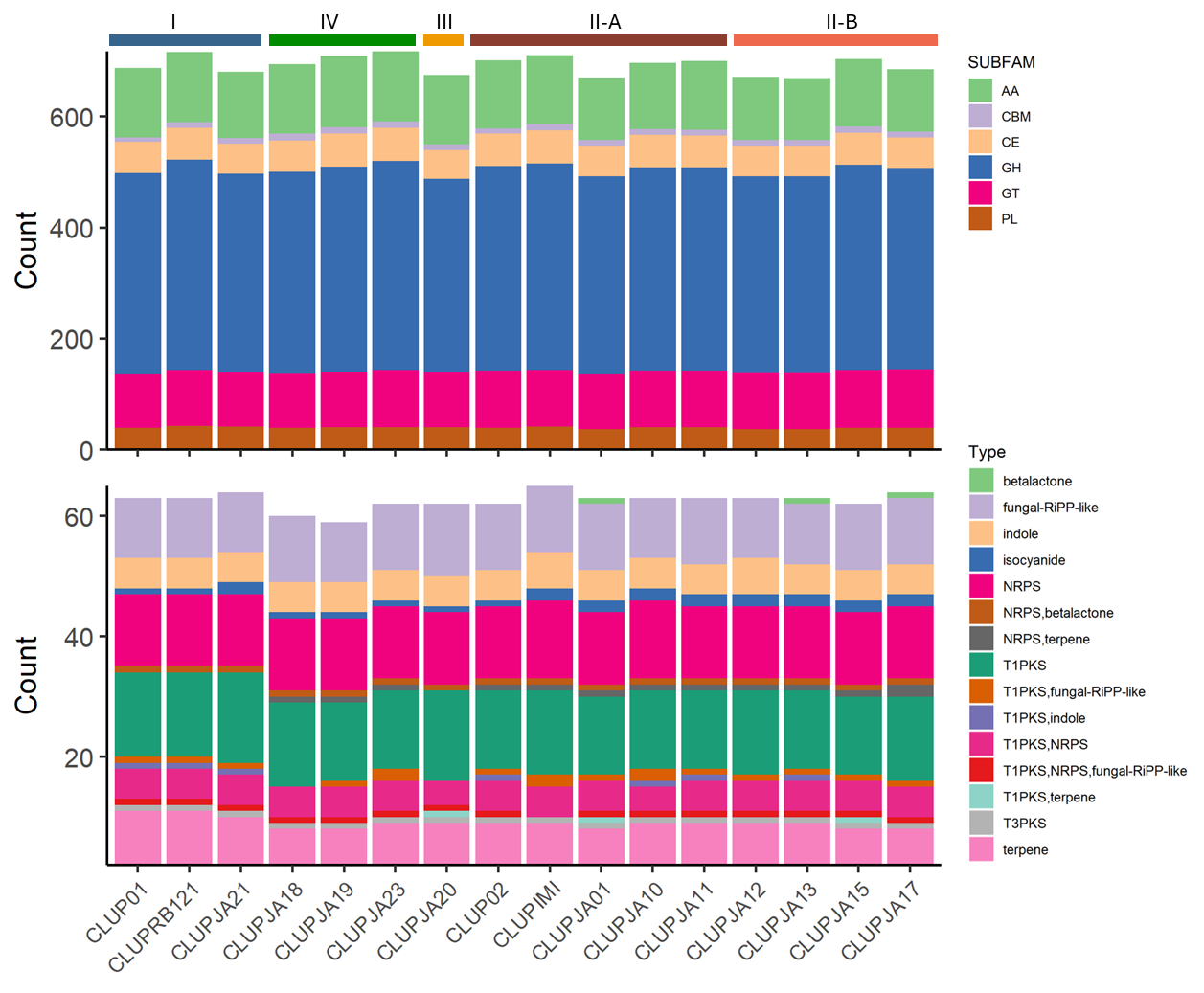


**Figure S9:** Total count and type of **(a)** secondary metabolite gene clusters and **(b)** carbohydrate-active enzymes (CAZymes). CAZyme types are divided into glycoside hydrolase (GH), glycosyl transferase (GT), auxiliary activity (AA), carbohydrate esterase (CE), carbohydrate-binding modules (CBM) and polysaccharide lyase activity (PL) categories.
