# Supplementary material for: Comparative Genomics Reveals Sources of Genetic Variability in the Asexual Fungal Plant Pathogen Colletotrichum lupini
Source: Mol Plant Pathol. 2024 Dec 13;25(12):e70039. doi: 10.1111/mpp.70039 (PMC11645255; doi:10.1111/mpp.70039)
Supplement: Supplementary file 10 — Figure S10. Gene distribution per gene category. CV indicates coefficient of variation and * indicates that distribution is significantly different form a Poisson distribution (p < 0.05). [file MPP-25-e70039-s015.docx]

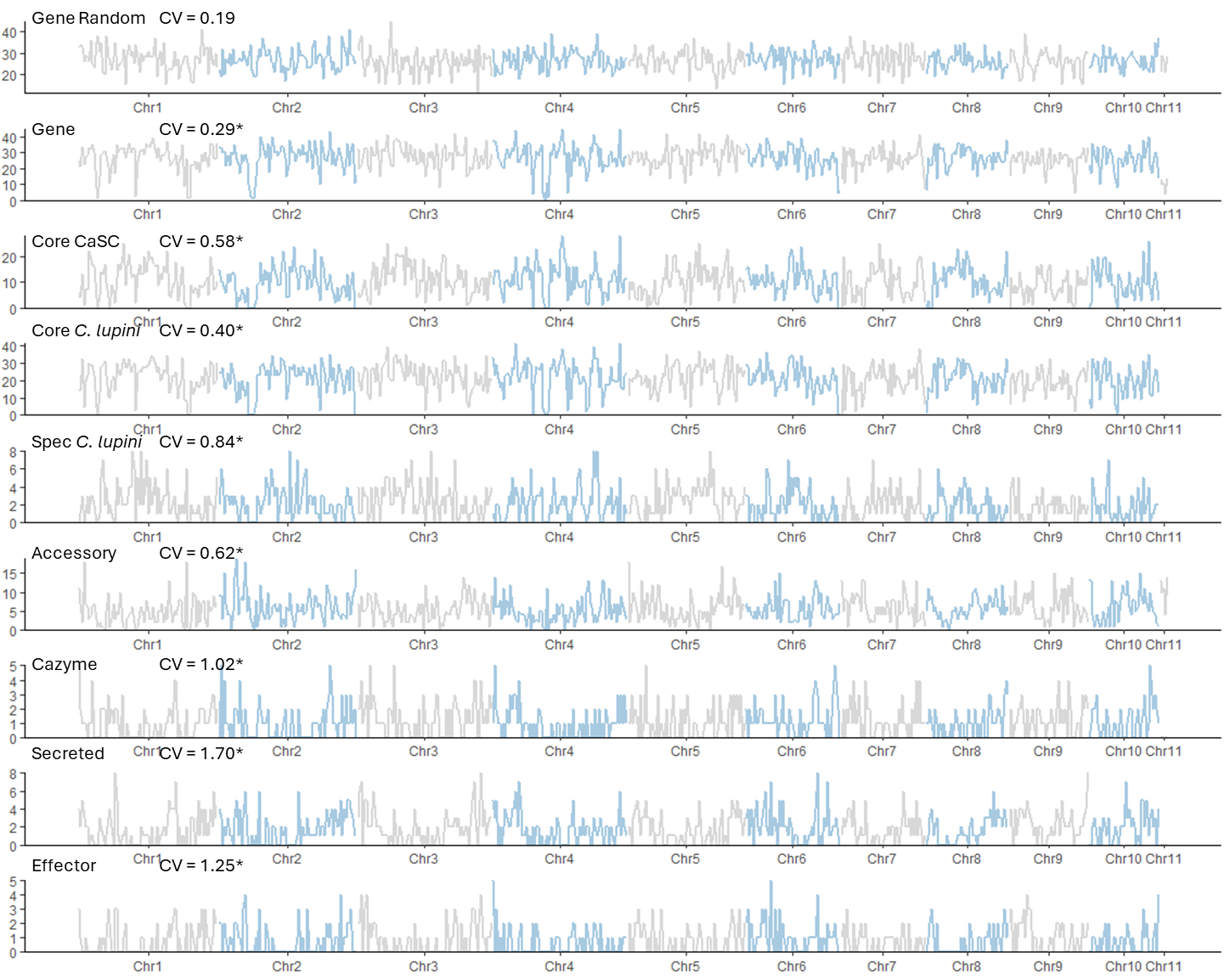


**Figure S10**: Gene distribution per gene category. CV indicates coefficient of variation and * indicates that distribution is significantly different form a Poisson distribution (p < 0.05).
