# Supplementary material for: Comparative Genomics Reveals Sources of Genetic Variability in the Asexual Fungal Plant Pathogen Colletotrichum lupini
Source: Mol Plant Pathol. 2024 Dec 13;25(12):e70039. doi: 10.1111/mpp.70039 (PMC11645255; doi:10.1111/mpp.70039)
Supplement: Supplementary file 11 — Figure S11. Proportion of genes per pangenome category closer than 10 kb to a transposable element (TE). [file MPP-25-e70039-s012.docx]

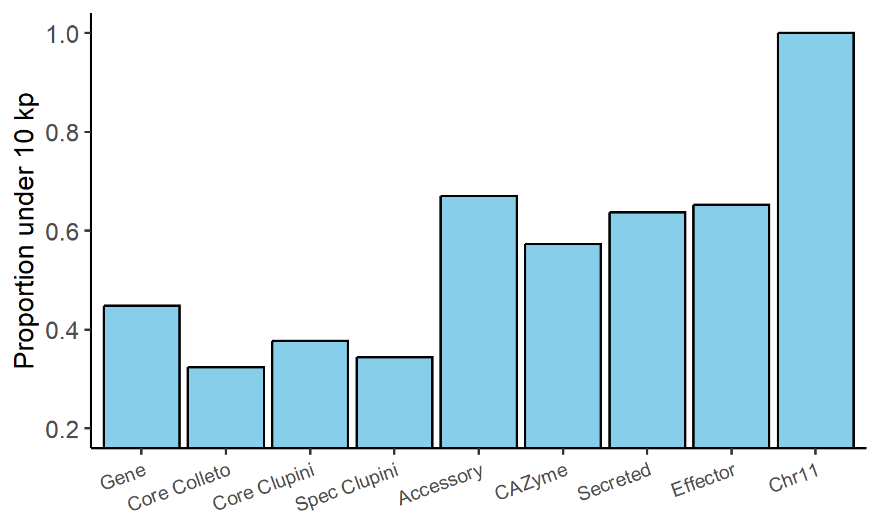


**Figure S11** Proportion of genes per pangenome category closer than 10 kb to a transposable element (TE).
