# Supplementary material for: Comparative Genomics Reveals Sources of Genetic Variability in the Asexual Fungal Plant Pathogen Colletotrichum lupini
Source: Mol Plant Pathol. 2024 Dec 13;25(12):e70039. doi: 10.1111/mpp.70039 (PMC11645255; doi:10.1111/mpp.70039)
Supplement: Supplementary file 12 — Figure S12. Selective sweep analysis and transposable element (TE) distribution across Colletotrichum lupini genome. (a) Genomic scans for selective sweeps for all 16 C. lupini isolates. CLR indicates composite‐likelihood‐ratio, and dashed line indicates CLR of 10, (b) analysis for lineage I isolates (n = 3), (c) analysis for lineage II isolates (n = 9) and (d) analysis for lineage IV isolates (n = 3). (e) Distribution of TEs across CLUP02 genome. [file MPP-25-e70039-s002.docx]

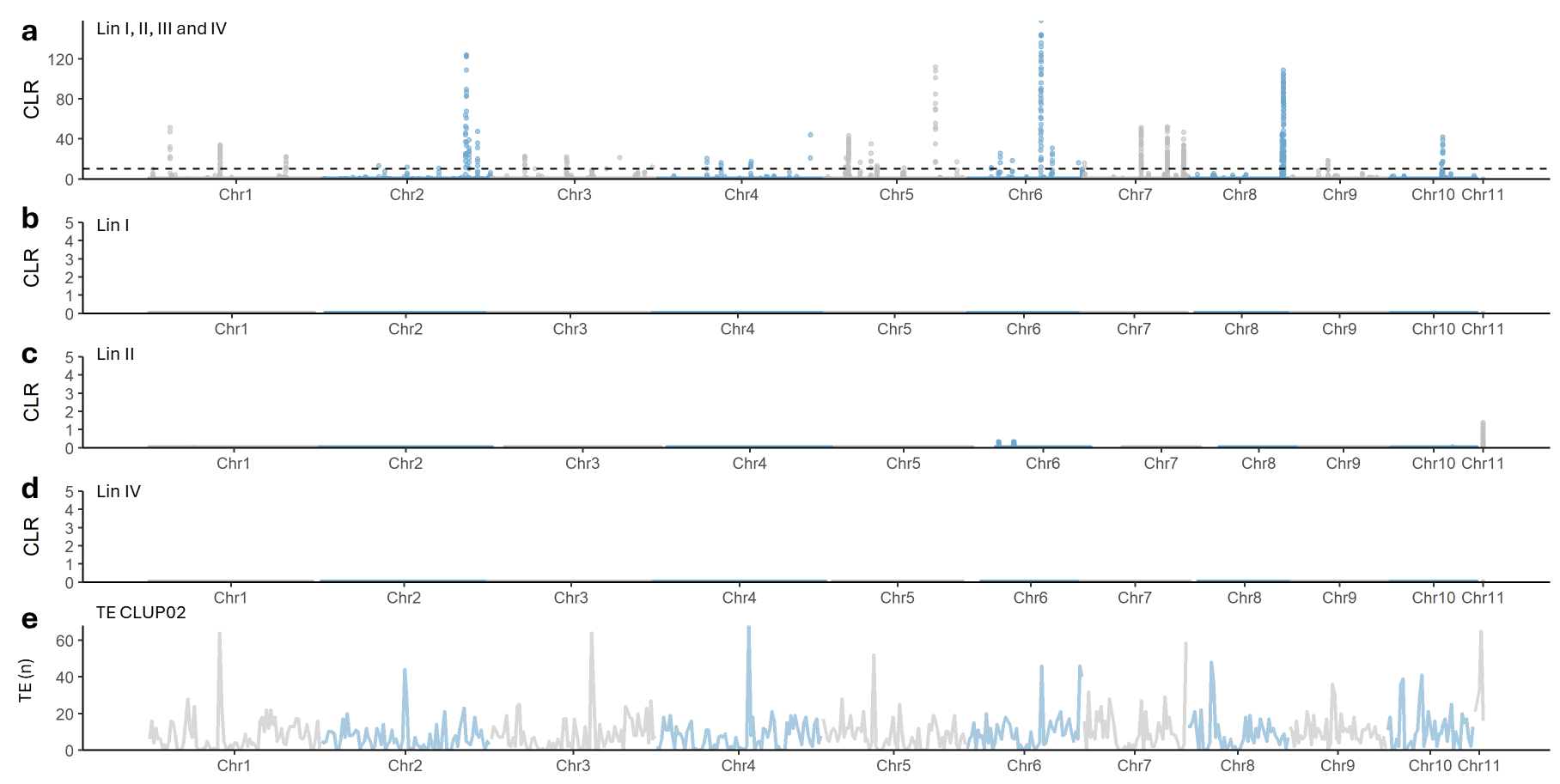
**Figure S12**: Selective sweep analysis and TE distribution across *Colletotrichum lupini* genome. **(a)** Genomic scans for selective sweeps for all 16 *C. lupini* isolates. CLR indicates composite likelihood ratio, and dashed line indicates CLR of 10, **(b)** analysis for lineage I isolates (n = 3), **(c)** analysis for lineage II isolates (n = 9) and **(d)** analysis for lineage IV isolates (n = 3). **(e)** Distribution of TEs across CLUP02 genome.
