# Supplementary material for: Comparative Genomics Reveals Sources of Genetic Variability in the Asexual Fungal Plant Pathogen Colletotrichum lupini
Source: Mol Plant Pathol. 2024 Dec 13;25(12):e70039. doi: 10.1111/mpp.70039 (PMC11645255; doi:10.1111/mpp.70039)
Supplement: Supplementary file 13 — Figure S13. Predicted effector clusters of species within the Colletotrichum acutatum species complex that are specific to or absent from all or some C. lupini lineages. Euclidian distance dendrograms are shown above and right of the plot. [file MPP-25-e70039-s006.docx]

**
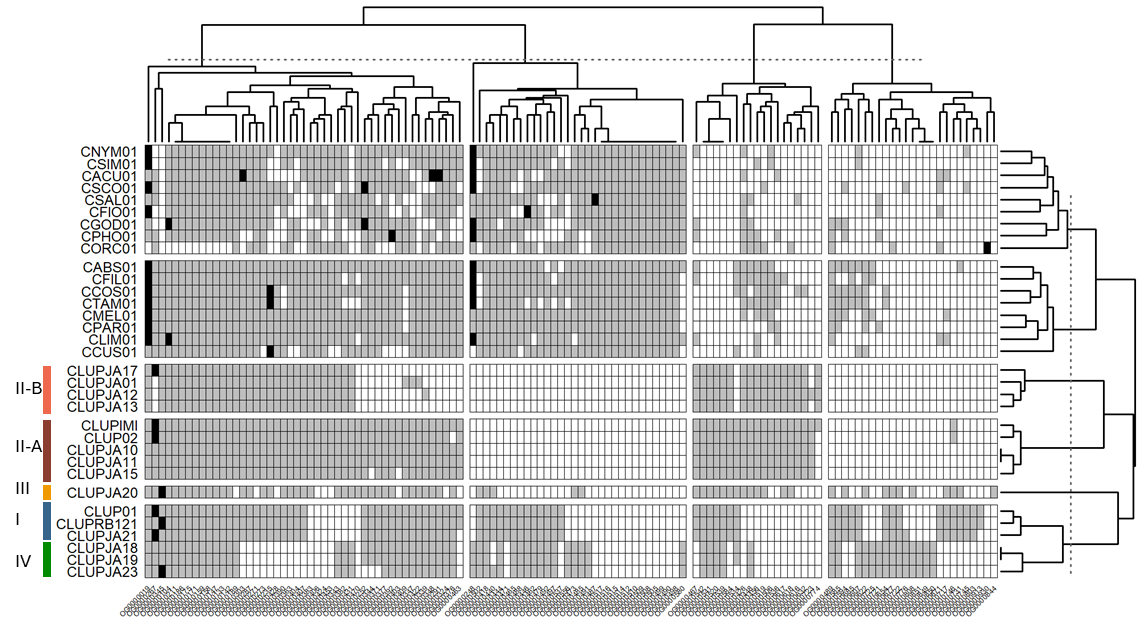
**

**Figure S13**: Predicted effector clusters of species within the *Colletotrichum acutatum* species complex that are specific to or absent from all or some *C. lupini* lineages. Euclidian distance dendrograms are shown above and right of the plot.
