# Supplementary figures and images for: Comparative Genomics Reveals Sources of Genetic Variability in the Asexual Fungal Plant Pathogen Colletotrichum lupini
Source: Mol Plant Pathol. 2024 Dec 13;25(12):e70039. doi: 10.1111/mpp.70039 (PMC11645255; doi:10.1111/mpp.70039)

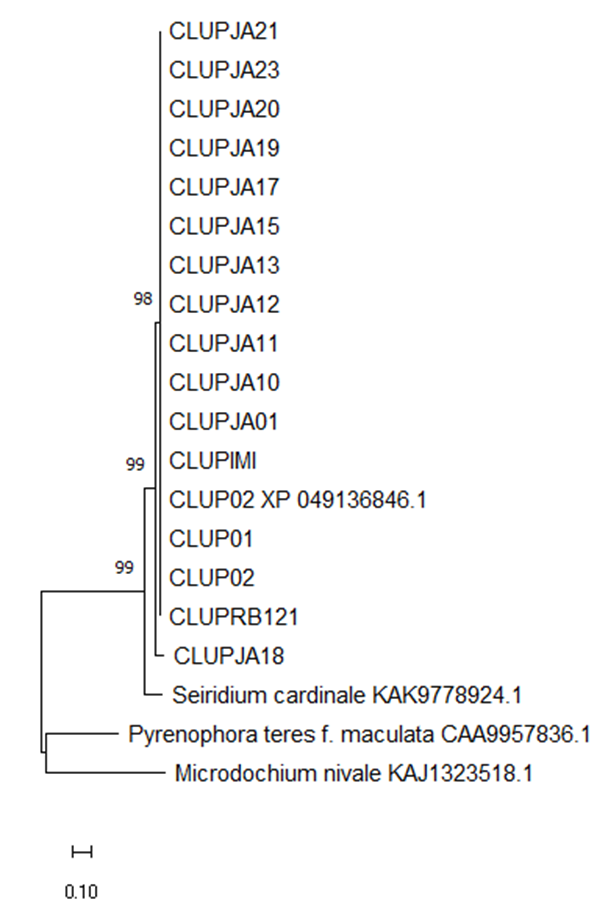


**Figure S14**: Maximum likelihood tree of OG0000520. Bootstrap support values (> 95) are given at each node.

Supplement: Supplementary file 14 — Figure S14. Maximum‐likelihood tree of OG0000520. Bootstrap support values ( > 95) are given at each node. [file MPP-25-e70039-s008.docx]
